# Application of multi-functional lactic acid bacteria strains in a pilot scale feta cheese production

**DOI:** 10.3389/fmicb.2023.1254598

**Published:** 2023-10-11

**Authors:** Christina S. Kamarinou, Olga S. Papadopoulou, Agapi I. Doulgeraki, Chrysoula C. Tassou, Alex Galanis, Nikos G. Chorianopoulos, Anthoula A. Argyri

**Affiliations:** ^1^Institute of Technology of Agricultural Products, Hellenic Agricultural Organization—DIMITRA, Athens, Greece; ^2^Department of Molecular Biology and Genetics, Democritus University of Thrace, Alexandroupolis, Greece; ^3^Laboratory of Food Microbiology and Hygiene, Department of Food Science and Technology, Faculty of Agriculture, Forestry and Natural Environment, School of Agriculture, Aristotle University of Thessaloniki, Thessaloniki, Greece; ^4^Laboratory of Microbiology and Biotechnology of Foods, School of Food and Nutritional Sciences, Department of Food Science and Human Nutrition, Agricultural University of Athens, Athens, Greece

**Keywords:** feta cheese, autochthonous cultures, multi-functional LAB, *Listeria monocytogenes*, sensory characteristics, organic acids, RAPD-PCR

## Abstract

Feta cheese is the most recognized Greek Protected Designation of Origin (PDO) product in the world. The addition of selected autochthonous lactic acid bacteria (LAB) strains to cheese milk as adjunct cultures is gaining more attention, since they can impact the nutritional, technological and sensory properties of cheeses, as well as improve the safety of the product. The aim of this study was to produce Feta cheese with enhanced quality and safety, and distinctive organoleptic characteristics by applying autochthonous lactic acid bacteria (LAB) with multi-functional properties as adjunct cultures. Feta cheeses were produced with the commercial lactococcal starter culture and the addition of 9 LAB strains (*Lactococcus lactis* SMX2 and SMX16*, Levilactobacillus brevis* SRX20*, Lacticaseibacillus paracasei* SRX10, *Lactiplantibacillus plantarum* FRX20 and FB1*, Leuconostoc mesenteroides* FMX3, FMX11, and FRX4, isolated from artisanal Greek cheeses) in different combinations to produce 13 cheese trials (12 Feta trials with the adjunct LAB isolates and the control trial). In addition, Feta cheese manufactured with FMX3 and SMX2 and control Feta cheese were artificially inoculated (4 log CFU/g) with *Listeria monocytogenes* (a cocktail of 4 acid or non-acid adapted strains). Cheese samples were monitored by microbiological and physicochemical analyses during ripening, and microbiological, physicochemical, molecular and sensory analyses during storage at 4°C. The results showed that after manufacture, the LAB population was *ca.* 9.0 log CFU/g at all samples, whereas during storage, their population declined to 6.5–7.0 log CFU/g. In the *Listeria* inoculated samples, *Listeria* was absent after 60 days (end of ripening) and after 90 days in the adjunct culture, and in the control trials, respectively. Moreover, the addition of selected strains, especially *Lcb. paracasei* SRX10, led to cheeses with desirable and distinctive organoleptic characteristics. Furthermore, randomly amplified polymorphic PCR (RAPD-PCR) molecular analysis confirmed that the multi-functional LAB strains were viable by the end of storage. Overall, the results of this study are promising for the use of autochthonous strains in various combinations with the commercial starter culture to satisfy industry requirements and consumer demands for traditional and high added value fermented products.

## 1. Introduction

Feta cheese, the “white gold” of Greece, is the most recognized traditional Greek food in the world. In 2002, Feta was approved as a Greek Protected Designation of Origin (PDO) product, according to Commission Regulation (EC) No. 1829/2002. Feta is a white soft cheese, ripened in brine and made exclusively of ewe’s milk or from a mixture of ewe’s and goat’s milk (up to 30%) (Commission Regulation (EC) No. 1829/2002). Many researchers have examined the microbial “fingerprint” of Feta PDO cheese, and their results showed that LAB are the predominant bacterial community with great diversity. For instance, Michailidou et al. (2021) reported that the genera *Lactococcus, Lactobacillus, Streptococcus* and *Leuconostoc* were the most prevalent in Feta cheeses during their shelf life. In addition, in a variety of commercial Feta cheese samples, *Lactococcus lactis*, *Streptococcus thermophilus* and *Lactobacillus delbrueckii* subsp*. bulgaricus* (commonly used as starter cultures) were the most abundant species detected (Papadakis et al., 2021; Papadimitriou et al., 2022). Papadakis et al. (2021) also reported that although the starter cultures were found in the highest abundance, the nonstarter LAB species diversity was significant, revealing a high degree of polymorphism at the strain and species level inside the cheese microbiota.

A common practice for cheese production when using pasteurized milk is the addition of commercial starter cultures to standardize the fermentation process and/or improve the sensory characteristics. However, these cultures are responsible for a flat organoleptic pattern rather than a rich traditional taste (Leroy and De Vuyst, 2004; Psoni et al., 2006), so the addition of selected autochthonous LAB strains to cheese milk as adjunct cultures is gaining more attention, as it is reflected in the recent scientific literature (de Souza and Dias, 2017; Gaglio et al., 2021; Bettera et al., 2022). These added microorganisms can release intracellular enzymes during ripening and storage, which mainly impact the nutritional, technological and sensory properties of cheeses (Escobar-Zepeda et al., 2016; Fox et al., 2017). For instance, González and Zarate (2012) studied a local Tenerife cheese produced with commercial starter and with the addition of a mix of indigenous strains (*Lc. lactis* subsp. *lactis* TF53, *Lactiplantibacillus plantarum* TF191 and *Leuconostoc mesenteroides* subsp. *mesenteroides* TF756). Their results showed that the cheese made with the autochthonous LAB had enhanced sensory features compared to the cheese made with the CS. Bancalari et al. (2020) evaluated the effect of an indigenous *Lacticaseibacillus paracasei* strain to be used as an adjunct culture for the production of an Italian semi-hard cheese. A clear differentiation in terms of aromatic profile, color, texture and sensorial perception was observed in the experimental cheese (with the *Lcb. paracasei* 4321) in contrast to the control. Finally, many researchers have used indigenous LAB strains as adjunct cultures to produce Feta-type cheese, and they observed an improvement in the textural and sensory characteristics (Michaelidou et al., 2003; Manolaki et al., 2006; Dimitrellou et al., 2014; Mantzourani et al., 2018).

Cheese is considered a safe food product, however, contamination with foodborne pathogens can occur at various stages of the food processing environment (Kousta et al., 2010). The most common foodborne pathogens present in white brined cheeses (WBC) are *Listeria monocytogenes*, enteropathogenic *Escherichia coli* O157:H7, *Yersinia enterocolitica* and *Salmonella* spp. *Staphylococcus aureus* (Bintsis and Papademas, 2002; Papadimitriou et al., 2022). Among those microorganisms, *L. monocytogenes* can be a serious concern since there are known outbreaks with fatalities originating from a variety of soft and semi-hard cheeses (Campagnollo et al., 2018). The presence of *L. monocytogenes* in dairy products indicates inadequate pasteurization of milk (Cadavez et al., 2019) or post-pasteurization contamination due to poor hygienic conditions during cheese-making (Kapetanakou et al., 2017). *L. monocytogenes* has been detected in WBC in different countries including Jordan (Osaili et al., 2012), Turkey (Arslan and Özdemir, 2008) and Greece (RASFF, 2022; Papadimitriou et al., 2022). In Jordan, the overall prevalence of *Listeria* spp. in WBC was 27.1%, with *L. monocytogenes* possessing 11.1% in the samples, while other species shared lower percentages (*Listeria grayi, Listeria innocua, Listeria ivanovii*, *Listeria seeligeri*, and *Listeria welshimeri*) (Osaili et al., 2012). In Turkey, the incidence of *Listeria* spp. in a homemade WBC was 9.2% for *L. monocytogenes*, while the other aforementioned species shared lower percentages (Arslan and Özdemir, 2008). In Greece, the presence of *L. monocytogenes* in Feta cheese was confirmed through shotgun metagenomics (Papadimitriou et al., 2022), while Rapid Alert System for Food and Feed (RASFF) issued an alert notification about the presence of *L. monocytogenes* in Feta cheese (RASFF 2022). However, LAB cultures can improve the safety of cheeses by inhibiting the growth of several pathogens. In fact, a large number of LAB strains have proven anti-listerial activity and many of them have been applied as adjunct cultures in cheese fermentation to control *L. monocytogenes* (Silva et al., 2018). Papadopoulou et al. (2018) studied the fate of artificially inoculated *L. monocytogenes* during ripening and storage of Feta cheese without or with the addition of *Lpb. plantarum* T571 as an adjunct culture and noticed that the added LAB strain inactivated the pathogen in shorter time compared to the control samples. Based on their results, it was evident that the pathogen cannot survive well in the Feta environment, however, it can still be present during the early storage of this product, posing a threat to the consumers. Prezzi et al. (2020) evaluated the effect of *Lacticaseibacillus rhamnosus* GG on the growth of and *L. monocytogenes* inoculated on the surface of Minas Frescal cheese during storage at 7°C. Their results showed a decrease of 1.1–1.6 log CFU/g in the population of *L. monocytogenes* after 21 days of storage. Morandi et al. (2019) investigated the effect of LAB strains on the growth of artificially inoculated *L. monocytogenes* on Gorgonzola rinds during ripening and noticed that, *Lc. lactis* (FT27, N16 and SV77) inhibited the growth of *L. monocytogenes* after 50 days of ripening.

Organic acids are present in dairy products due to bacterial growth during the fermentation processes and as a result of normal biochemical metabolism (breakdown of milk components, i.e., proteins, fat, and lactose) (Izco et al., 2002). The most common organic acids in cheeses are lactic, acetic, and propionic acids (Hayaloglu, 2016). The level of each organic acid can be affected by the starters, the adjunct cultures and the non-starter lactic acid bacteria (NSLAB) present in the cheeses (Skeie et al., 2001; Manolaki et al., 2006). Overall, the presence of organic acids in dairy products is important for both flavor and preservation. In more detail, organic acids help to lower the pH of dairy products, which inhibits the growth of harmful bacteria and other spoilage microorganisms, thus extending their shelf life. They also contribute to the characteristic flavor and texture of dairy products (Jo et al., 2018; Pisano et al., 2020; Ahmed et al., 2021).

Based on the above, the aim of this study was to develop Feta cheese with enhanced quality and safety, as well as distinctive organoleptic characteristics. Thus, 9 indigenous LAB strains (in mono and mixed combinations) previously isolated from Greek cheeses and characterized with good technological (ability to produce β-galactosidase, partial proteolysis, no diacetyl production, and exopolysaccharides (EPS) production and functional characteristics (good survival rates at low pH and at bile salts) and/or attributed good organoleptic properties when used as adjunct cultures *in situ* (yogurt) (Kamarinou et al., 2022), were incorporated as adjunct cultures in the production of 12 different Feta trials, to evaluate their effect on the microbiological, physicochemical and sensory characteristics of the cheeses, during ripening and storage compared to the control trial. The strains that exhibited good technological and functional properties but did not attribute positive organoleptic properties to yogurt (Kamarinou et al., 2022) were applied only as mixed cultures. Furthermore, in selected cheese trials, the changes in organic acids were evaluated using high-performance liquid chromatography (HPLC), whereas the recovery rate of the added LAB strains was investigated using Random Amplified Polymorphic (RAPD-PCR). Finally, in order to monitor the survival of the pathogen *L. monocytogenes*, Feta cheese produced with a co-culture of *Leuconostoc mesenteroides* FMX3 and *Lactococcus lactis* SMX2 with *in vitro* anti-listerial activity (Kamarinou et al., 2022) was artificially inoculated with a four (4) strain cocktail of *L. monocytogenes*.

## 2. Materials and methods

### 2.1. Preparation of LAB adjunct cultures

Nine LAB strains (*Lc. lactis* SMX2, SMX16, *Levilactobacillus brevis* SRX20, *Lpb. plantarum* FRX20, FB1*, Ln. mesenteroides* FRX4, FMX3, FMX11, and *Lcb. paracasei* SRX10), which have been previously isolated from traditional Greek cheeses (Kamarinou et al., 2022), were used as adjunct cultures in Feta cheese production in various combinations as mono or co- cultures (Table 1). The different trials were selected by preliminary experiments producing yogurt with ewe’s and goat’s milk (in a ratio of 80:20) to mimic the consequent Feta cheese substrate and implementing sensory evaluation, according to the procedure described by Kamarinou et al., 2022. After this procedure, seven strains (SMX16, SRX20, FRX20, FB1, FRX4, FMX11, and SRX10) were selected to be applied as adjuncts in Feta monocultures in mono- or co-cultures (combinations of 2 or 3 strains), due to their contribution to the aforementioned yogurts sensory profiles (see Supplementary Figure S1). The co-cultures were developed by combining only different microbial species. Additionally, two (2) strains (SMX2, FMX3) with anti-listerial activity *in vitro* (Kamarinou et al., 2022), were applied as adjuncts in Feta in mono- cultures or in combination. The strains were revived from a stock culture stored at −80°C and were grown aerobically in the appropriate culture media, i.e., de Man, Rogosa and Sharpe broth (MRS) (LAB094, LABM, Lancashire, United Kingdom) for lactobacilli and M17 broth (BK012HA, Biokar Diagnostics, Allonne, France) for lactococci, at 30°C and 37°C for 24 h, respectively. Fresh monocultures of the isolates were harvested by centrifugation (5,000 g, 10 min, 4°C), washed twice with ¼ strength Ringer’s solution (LMNCM0191K, Neogen Corporation, Michigan, USA) and resuspended in 10 mL Ringer’s solutions. To prepare the cocktail of the strains, the cells were mixed in the appropriate ratio (Table 1). Inoculum size was confirmed before use by the serial dilution method on MRS ISO agar (NCM0190, Neogen Corp.) and M17 Agar (4017194, Biolife, Milano, Italy).

**Table 1:** Combinations of the adjunct LAB isolates and the commercial starter culture used for the manufacture of Feta cheese.

Feta case	Starter/adjunct culture combination	Ratio %
Feta 1	Commercial Starter culture *Lacticaseibacillus paracasei* SRX10	50:50
Feta 2	Commercial Starter culture *Lactiplantibacillus plantarum* FRX20	50:50
Feta 3	Commercial Starter culture *Leuconostoc mesenteroides* FRX4	50:50
Feta 4	Commercial Starter culture *Lactococcus lactis* SMX2	50:50
Feta 5	Commercial Starter culture *Leuconostoc mesenteroides* FMX3	50:50
Feta 6	Commercial Starter culture *Lactobacillus plantarum* FB1, *Levilactobacillus brevis* SRX20	50:25:25
Feta 7	Commercial Starter culture *Lacticaseibacillus paracasei* SRX10, *Levilactobacillus brevis* SRX20	50:25:25
Feta 8	Commercial Starter culture *Leuconostoc mesenteroides* FMX11, *Lactiplantibacillus plantarum* FB1, *Lacticaseibacillus paracasei* SRX10	50:17:17:17
Feta 9	Commercial Starter culture *Lactococcus lactis* SMX16, *Leuconostoc mesenteroides* FRX4	50:25:25
Feta 10	Commercial Starter culture *Lactiplantibacillus plantarum* FB1, *Lacticaseibacillus paracasei* SRX10	50:25:25
Feta 11	Commercial Starter culture *Leuconostoc mesenteroides* FMX11, *Levilactobacillus brevis* SRX20, *Lactiplantibacillus plantarum* FRX20	50:17:17:17
Feta 12	Commercial Starter culture *Leuconostoc mesenteroides* FMX3, *Lactococcus lactis* SMX2	50:25:25
Control	Commercial Starter culture	100

### 2.2. Pilot-scale feta cheese production

Feta cheeses were produced at our industrial partner, a Greek dairy industry located in Trikala, Greece (Hellenic Dairies S.A, Trikala, Greece). The cheese production followed the typical industrial procedure. In brief, pasteurized ewe’s and goat’s milk (in a ratio of 80:20) was inoculated with the commercial starter culture (Choozit™ MA 11 LYO 250 Danisco Culture Unit - DCU, Danisco, Copenhagen, Denmark) containing *Lc. lactis* subsp. *lactis* and *Lc. lactis* subsp. *cremoris* (control trial), or with the addition of the selected LAB strains in mono and co- cultures along with the commercial starter culture in a ratio of 1:1, as shown in Table 1. The inoculum of all added cultures in the pasteurized milk was approximately 7 log CFU/mL. After milk coagulation, the curd was cut and transferred to molds, where dry salt was added and the molds were turned upside down at appropriate times for 24 h to ensure even drainage. On the following day, the cheese pieces with pH 4.7 ± 0.04 (*ca.* 2 Kg from each mold) were placed in metal vessels of 16 kg, brine (7% w/v NaCl) was added and ripening followed. Ripening took place in two periods: (a) 7 days at 18°C (1^st^ ripening), and (b) 53 days at 4°C (2^nd^ ripening). After ripening, cheese of *ca.* 2 kg (pH 4.4 ± 0.1) was placed in plastic containers and fresh brine (7% w/v NaCl) was added. Samples were then stored at 4°C for 120 days (expiration date of commercial products). In total, 13 different trials were produced (12 Feta trials with the adjunct LAB isolates and the control trial-Table 1). The experiment was repeated twice, i.e., 2 different seasonal batches (September and January) with 3 replicates studied at every time point for each trial. The samples after production were coded and stored in the appropriate temperatures throughout ripening and storage. To ensure the randomization of the samples, an online computer software (Social Psychology Network, Middletown, CT, United States) was used, which is available at “https://www.randomizer.org/”.

### 2.3. Preparation of acid and non-acid adapted *Listeria monocytogenes* cells

A cocktail of four *L. monocytogenes* strains was used to artificially inoculate Feta cheese, as described below. The strains FMCC-B-129 and FMCC-B-133 were provided by the laboratory of Food Microbiology and Biotechnology of the Agricultural University of Athens and the strains DSM15675 and DSM19094 were purchased from the German Collection of Microorganisms and Cell Cultures (DSMZ, Braunschweig, Germany). The strains were revived from a stock culture stored at −80°C and grown aerobically in 10 mL Tryptic Soy Broth (TSB) (LAB004, Neogen Corp.) and incubated at 37°C for 18 h. A subculture of each strain was prepared in fresh 10 mL TSB supplemented with 0.6% yeast extract (MC001, Neogen Corp.) (TSB-YE) and incubated at 37°C for 18 h. 100 *μ*L of each overnight culture of *L. monocytogenes* was inoculated in fresh 10 mL TSB-YE broth and incubated at 37°C, until reaching a 0.15 optical density (OD) (Cataldo et al., 2007). All the strains reached a population of *ca.* 6 log CFU/mL in the aforementioned OD. The OD was monitored using a microplate reader (SpectraMax Plus 384 Microplate Reader, Molecular Devices, Sunnyvale, VA, United States) by measuring the absorbance at 600 nm. Then, the cells were harvested by centrifugation (5,000 g, 10 min, 4°C) and each pellet were resuspended in fresh TSB-YE broths with pH adjusted to 5.0 using 85% lactic acid (acid adapted) or in TSB-YE broths with pH adjusted to 7.0 (non-acid adapted/control) and subsequently incubated at 37°C for 1 and 3 h (Cataldo et al., 2007). In order to determine the acid tolerance response (ATR), the cells were harvested by centrifugation (5,000 g, 10 min, 4°C), resuspended in fresh TSB-YE broths with pH adjusted to 3.5, and subsequently incubated for 2 h at 37°C, while their survival was determined by enumeration on Tryptic Soy Agar (TSA) (BK047HA, Biokar Diagnostics) plates (24–48 h at 37°C). Acid response (AR) was determined by centrifuging each overnight culture of *L. monocytogenes* in TSB-YE (5,000 g, 10 min, 4°C). Then bacteria were resuspended in TSB-YE acidified to pH 3.5 for 2 h at 37°C and their survival was determined by enumeration on TSA plates (Cataldo et al., 2007).

Τhe *Listeria* strains (acid or non-acid adapted) were mixed in equal volumes to achieve a cocktail culture with a final population of 4 log CFU/g in the cheese. Each of the *Listeria* strains (acid or non-acid adapted) population, as well as the inoculum size of the cocktail culture were confirmed before use by the serial dilution method on Palcam Agar (BK145HA, Biokar Diagnostics) with a Palcam selective supplement (BS00408, Biokar Diagnostics).

### 2.4. Feta cheese inoculation with *Listeria monocytogenes* strains

The day after Feta cheese production (day 1), samples without (control samples) or with the addition of *Ln. mesenteroides* FMX3 and *Lc. lactis* SMX2 (Feta L12, Table 2), were artificially inoculated with the pathogen in the Institute of Technology of Agricultural Products Laboratory (Hellenic Agricultural Organization - DIMITRA, Lycovrissi, Greece). Each cheese piece of 2 kg (22 × 10 × 10 cm) was inoculated with *L. monocytogenes* (cocktail of four strains, acid or non-acid adapted, Table 2) using sterilized syringes, to reach a final population of 4 log CFU/g in the cheese. The inoculum was injected in each of the four 22 × 10 sides of the cheese pieces. In this respect, the syringe was slowly immerged in every 10 cm^2^ of each side in a depth of 3.5 cm, releasing 250 *μ*L of inoculum (6 log CFU/mL). Consequently, the exact procedure of cheese ripening as with the non-inoculated (without *L. monocytogenes*) cheeses was followed.

**Table 2:** Feta cheese trials artificially inoculated with acid or non-acid adapted *Listeria monocytogenes* (4 strain cocktail).

Feta case (Code names)		Starter/adjunct culture combination	Ratio (%)	*Listeria monocytogenes* Inoculum
Batch 1	Batch 2			
FLA1	FLA2	Commercial Starter culture *Ln. mesenteroides* FMX3 *Lc. lactis* SMX2 (Feta L12)	50:25:25	Acid adapted cells Cocktail of 4 strains FMCC-B129 FMCC-B133 DSMZ15675 DSMZ19094
CLA1	CLA2	Commercial Starter culture	100	
FLN1	FLN2	Commercial Starter culture *Ln. mesenteroides* FMX3 *Lc. lactis* SMX2 (Feta L12)	50:25:25	Non-acid adapted cells Cocktail of 4 strains FMCC-B129 FMCC-B133 DSMZ15675 DSMZ19094
CLN1	CLN2	Commercial Starter culture	100	

### 2.5. Microbial enumeration

The microbial analyses of the Feta cheese samples (samples without the pathogen) were carried out at six time points (1, 60, 90, 120, 150 and 180 days after cheese production). The samples inoculated with *L. monocytogenes* (Feta L12 and the control) were examined at 12 time points (1, 5, 8, 15, 22, 36, 50, 60, 90, 120, 150 and 180 days after cheese production). In brief, 25 g of triplicate Feta cheese samples (from each batch) were aseptically added to 225 mL of sterilized ¼ Ringer’s solution and subjected to serial dilutions in the same diluent. 0.1 or 1 mL samples were spread or poured on the following agar media; Plate Count Agar (4021452, Biolife) for Total Aerobic Viable Counts (TVC), incubated at 30°C for 48–72 h, MRS ISO Agar overlaid with the same medium and incubated at 30°C for 48–72 h for the enumeration of mesophilic LAB, M17 Agar overlaid with the same medium incubated at 37°C and 42°C for 48–72 h for the enumeration of lactococci, Baird Parker Agar (LAB285, LABM) with Egg Yolk Tellurite (X085, LABM) for coagulase-positive staphylococci incubated at 37°C for 48 h, Violet Red Bile Glucose Agar (402.85, Biolife) for *Enterobacteriaceae* counts overlaid with the same medium and incubated at 37°C for 18–24 h, Xylose Lysine Deoxycholate (CM0469, Oxoid) for *Salmonella* spp. incubated at 37°C for 16–18 h, Palcam Agar with Palcam selective supplement for the enumeration of *L. monocytogenes* incubated at 37°C for 24 and 48 h (to lower the detection limit of the pathogen to 1.0 log CFU/g, 1 mL of the samples’ first decimal dilution was spread onto three Petri dishes). To ensure the absence of *Salmonella* spp. and *L. monocytogenes* enrichment was applied in the samples according to ISO 6579-1:2017 and ISO 11290-1:2017, respectively. (International Organizational for Standardization, 2017a,b).

### 2.6. Physicochemical analysis

The pH of cheese samples was measured at the same time intervals as with the microbiological analysis by immersing the penetrating electrode (FC200B, HANNA instruments, Woonsocket, RI, USA) in the cheese samples. pH measurements were carried out using a digital pH-meter (HI2211, pH/ORPMeter, HANNA instruments).

The cheeses (1^st^ batch) were also analyzed in the premises of Hellenic Dairies S.A. for their physicochemical characteristics, i.e., protein, fat, moisture and sodium chloride, during storage using FoodScan™ Lab (Routine Analysis Software, FOSS, Hillerød, Denmark), with three measurements per sample being obtained each time.

### 2.7. Sensory evaluation

A group of 10 people, previously trained in evaluating dairy products (staff from the Institute of Technology of Agricultural Products of Hellenic Agricultural Organization - DIMITRA and the industry), were selected to form the sensory panel. The selection criteria for the sensory panel are thoroughly explained in the previous work by Papadopoulou et al. (2018). The evaluation was carried out under artificial lighting conditions in individual booths in the sensory analysis room located in the Institute of Technology of Agricultural Products. All panelists provided consent prior to their participation in the study. For sensory evaluation, non-pathogen inoculated cheeses from all Feta trials were used. Initially, the overall quality in terms of like or dislike was evaluated (total appearance, total aroma, total taste, and total texture) and then the specific sensory attributes were examined. The specific indicators of each sensorial attribute included appearance, i.e., white color, cracks, and holes on cheese; aroma; i.e., butter, and acid, taste; i.e., bitter, acid, salty, sweet, and texture; i.e., consistency, graininess, and hardness. Data were obtained through a 10-cm hedonic scale, where the scale direction was from left to right with increasing intensities, i.e., weak to strong, little to much, etc. (Papetti and Carelli, 2013).

### 2.8. Determination of organic acids by high-performance liquid chromatography

A high-performance liquid chromatography (Agilent 1200 series, Agilent Technologies, Palo Alto, CA, USA) was performed to determine the concentration of organic acids in six selected cheese trials. Duplicate samples (36) of the aforenoted trials were examined at different time points (0, 60, 90, 120, 150 and 180 days). To prepare the samples for HPLC analysis, the method of Argyri et al. (2011) was followed. In brief, 2 g of each cheese sample was homogenized with 4 mL sterile deionized water for 2 min, centrifuged (9,000 × g, 5 min, 4°C) and then 20 *μ*L of trifluoroacetic acid (Trifluoroacetic Acid for UV, PanReac AppliChem, Darmstadt, Germany) was added at 2 mL of the supernatant. Samples were again centrifuged at the same conditions and the collected supernatant was filtered through a 0.22 filter (Millipore PVDF Syringe filter/L0.22 *μ*m). For HPLC analysis, 20 *μ*L of the sample was injected into the injection port. Elution was carried out using a 0.009 N H_2_SO_4_ solution as the mobile phase, with a flow rate of 0.7 mL/min and an oven temperature of 65°C (Skandamis and Nychas, 2001). The separation was performed isocratically using a cation exchange Repromer H column (9 *μ*m 300 × 7.8 mm, Dr. Maisch GmbH, Germany). The HPLC system was equipped with a G1314A Variable Wavelength Detector and a Rheodyne HPLC manual injector (Model 7,010). The UV Absorbance was monitored at a wavelength of 210 nm and the Agilent ChemStation software (Agilent ChemStation Software B.02.01.SR1 Revision, Agilent Technologies, Inc., Santa Clara, CA, USA) was used for data acquisition and processing. Αs reference substances, solutions (1 to 0.001% w/v) of citric, lactic, tartaric, acetic, malic, propionic, oxalic, succinic and formic acids (HPLC grade) were used.

### 2.9. LAB survival and strain differentiation using RAPD -PCR

At the end of storage (180 day), a total of 58 LΑΒ strains were randomly collected from the highest countable dilution (≥6 log CFU/g) of MRS or M17 agar plates of 5 Feta cheese trials (Feta 1, 9, 10, 11 and 12) according to Papadopoulou et al. (2018). All the recovered isolates were stored at −80°C in MRS or M17 broths supplemented with 30% (v/v) glycerol until use. DNA was extracted according to Cocolin et al. (2007). RAPD-PCR was performed using the universal oligonucleotide primer M13 (5′- GAGGGTGGCGGTTCT-3) according to Giraffa et al. (2000). PCR amplicons were stained with GelRed (6X GelRed® Prestain Loading Buffer with Tracking Dye, Biotium, California, USA) and visualized on agarose gel (2%) using a Gel Doc System (Gel Doc Go Imaging System, Bio-Rad, Hercules, CA, USA). As a DNA molecular weight marker, a 1 kbp plus DNA Ladder (New England Biolabs, Ipswich, MA, USA) was included. The obtained profiles of the recovered LAB strains were compared with the known LAB profiles (commercial cheese starters and adjunct LAB strains) (Supplementary Figure S2).

### 2.10. Statistical analysis

Data were indicated as means; mean ± SD of three replicates × two different batches. Microbiological, physicochemical, sensory and HPLC results were analyzed for statistical significance with one-way analysis of variance (ANOVA) (StataMP17, StataCorp LLC, Texas, United States). Significance was established at *p* < 0.05. *Post hoc* analysis- Tukey’s HSD test was performed to determine significant differences among results. Additionally, data mining and interpretation of the sensory data and the HPLC data were performed using the online platform MetaboAnalyst v3.0″ (McGill University-Xia Lab, Montreal, QC, Canada, www.metaboanalyst.ca, access date: 20/10/2022). Two datasets were created (i) using the sensory scores (X variables) and the different storage days (60–180 day) (Y variables) of the 13 cheese trials and (ii) the organic acid compounds after HPLC analysis (X variables) and the different storage days (1–180 day) (Y variables) of the six cheese trials. The data sets were uploaded to the online platform and first transformed via scaling (autoscale) and then data analytics (partial least squares–discriminant analysis [PLS-DA] and hierarchical cluster analysis, i.e., heatmaps and dendrograms) were performed on the datasets.

## 3. Results and discussion

### 3.1. Microbial enumeration of the 13 feta trials

**Figure 1:**
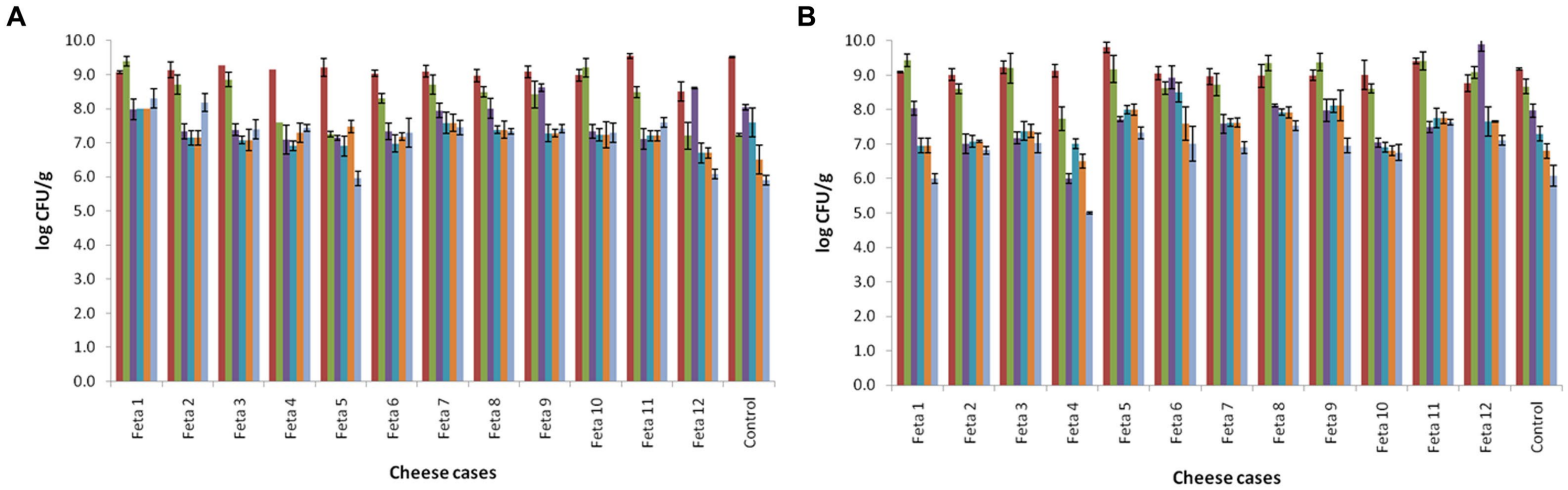
Population of mesophilic lactobacilli **(A)** and lactococci **(B)** of Feta cheese trials (according to Table 1), on the 1^st^ (■1d), 60^th^ (■60d), 90^th^ (■90d), 120^th^ (■120d), 150^th^ (■150d) and 180^th^ day (■180d) after cheese production. The bars represent the mean values ± standard deviations.

**Figure 2:**
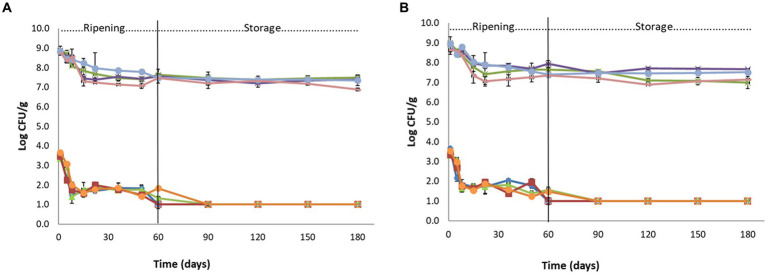
Population of *L. monocytogenes* (♦): FLA1, (■): FLN1, (▲): CLA1, (●): CLN1 and population of TVC: (⁎): FLA1, (●): FLN1, (x): CLA1, (**-**): CLN1 in Feta cheeses (Feta case L12 and the control) during ripening and storage (Batch 1 - **A**, Batch 2 - **B**). Open symbols indicate absence of *L. monocytogenes* after applying enrichment method. The bars represent the mean values ± standard deviations. FLA: Feta L12 inoculated with acid adapted *Listeria* strains, FLN: Feta L12 inoculated with non-acid adapted *Listeria* strains, CLA: Control Feta inoculated with acid adapted *Listeria* strains, CLN: Control Feta inoculated with non-acid adapted *Listeria* strains.

The changes in the populations of mesophilic LAB and lactococci in the 13 Feta trials during ripening and storage are presented in Figures 1A,B, respectively. The population of mesophilic LAB on the 1^st^ day after cheese production was found between 8.5–9.2 log CFU/g in the trials produced with the adjunct cultures (Feta 1–12), while for the control, the population was found at 8.3 ± 0.02 log CFU/g (*p* < 0.05). At the end of ripening (60th day), the population in all trials decreased by approximately 0.5 log CFU/g. During storage, an additional decrease was observed in the population of mesophilic LAB in all Feta trials (*p* < 0.05). In more details, Feta trials with the adjunct cultures displayed a population of 6.0 to 8.3 log CFU/g, while the control showed a population of 5.9 ± 0.14 log CFU/g at the end of storage (*p* < 0.05). Regarding the lactococci, their population in all Feta trials the 1^st^ day after cheese production was found between 8.7–9.0 log CFU/g (*p* < 0.05). During ripening, their population decreased to levels of 7.5–8.9 log CFU/g, depending on the trial (*p* < 0.05) (Figure 2). During storage, an additional decrease was observed, where the population levels in Feta trials 1–12 decline to final population of 6.5 to 8.1 log CFU/g (depending on the trial), while in the control, a lower population was observed (6.1 ± 0.31 log CFU/g) (*p* < 0.05). TVC followed the population of the dominant microbiota for each trial, i.e., mesophilic LAB and/or lactococci and thus presented similar counts (data not shown). Finally, the rest of the examined microbiota, i.e., *Enterobacteriaceae*, staphylococci, yeasts and molds, were always below the detection limit of the enumeration method. Pathogenic microorganisms, such as *L. monocytogenes* and *Salmonella* spp. were absent after enrichment.

The reduction trend of LAB (lactococci and lactobacilli) during ripening and storage is in accordance with previous studies on pilot Feta cheese production. For instance, Papadopoulou et al. (2018) studied the microbiota of an industrial Feta produced with the addition of *Lpb. plantarum* T571 and observed that during cold storage of Feta, the population of mesophilic lactobacilli and lactococci decreased in all samples. In the same study, it was also observed that the addition of the adjunct culture did not influence the level of lactobacilli and lactococcci counts throughout ripening and storage, a result that has also been found in other studies concerning Feta cheese (Dimitrellou et al., 2014; Papadopoulou et al., 2018). On the contrary, other researchers noted that in Feta-type cheeses that were produced with the use of *Lcb. casei* ATCC 393 as adjunct culture, the population of lactobacilli was significantly increased after 120 days of storage, while the population of lactococci did not significantly differ among the samples (Terpou et al., 2018a). The absence of *Enterobacteriaceae*, staphylococci, *Listeria* spp., *E. coli* and yeasts and molds in the present study verifies the high hygienic conditions during the production of cheese.

### 3.2. Feta inoculated with *Listeria monocytogenes*

Before Feta inoculation, the survival of the *Listeria* strains after acid adaptation (pH 5) for 1 and 3 h and subsequent incubation at lethal pH (3.5) for 2 h was evaluated. Among the 4 tested strains, the survival of strain FMCC-B-129 showed a strong ATR for both 1 and 3 h of incubation at moderate pH (pH 5), which subsequently resulted in elevated survival (89.9 and 91.2% of the bacterial population, respectively) after exposure to lethal pH. However, the other 3 strains exhibited a better survival rate (81.5–84.9%) only after 1 h of exposure to moderate pH, so the 1 h of acid adaptation was selected for this study. Regarding the AR, results demonstrated that the survival of the strains was very low (<30%), except for the strain FMCC-B-129 which showed a moderate survival (52%). The changes in the population of *L. monocytogenes* and TVC during ripening and storage in *Listeria* inoculated cheeses (Feta L12 and the control samples) are presented in Figure 2.

After the inoculation of the fresh cheese (day 1) with the acid and non-acid adapted *Listeria* strains, the population of the pathogen was estimated at approximately 3.30–3.65 log CFU/g, depending on the trial (*p* < 0.05). During ripening, it was observed that the *Listeria* population had a decreasing trend at all samples (the *p*-values for the 1st, 5th, 36th, 50th, and 60th days were found to be less than 0.05, however, for the 8th, 15th, and 22nd days after production, the p-values were *p* ≥ 0.05), but this decrease was more intense at Feta trial 12 inoculated with both acid and non-acid adapted *Listeria* strains at both batches (Table 2). In more detail, until the 50^th^ day of ripening, the population of *L. monocytogenes* decreased in all Feta L12 trials (acid and non-acid adapted strains) by 1.4–2.3 log CFU/g, depending on the trial. At the 60^th^ day (end of ripening), the pathogen was not detected after enrichment in all trials of Feta L12, while the population of the pathogen in the control trials was estimated at 1.3–1.8 log CFU/g (Figures 2A,B).

**Figure 3:**
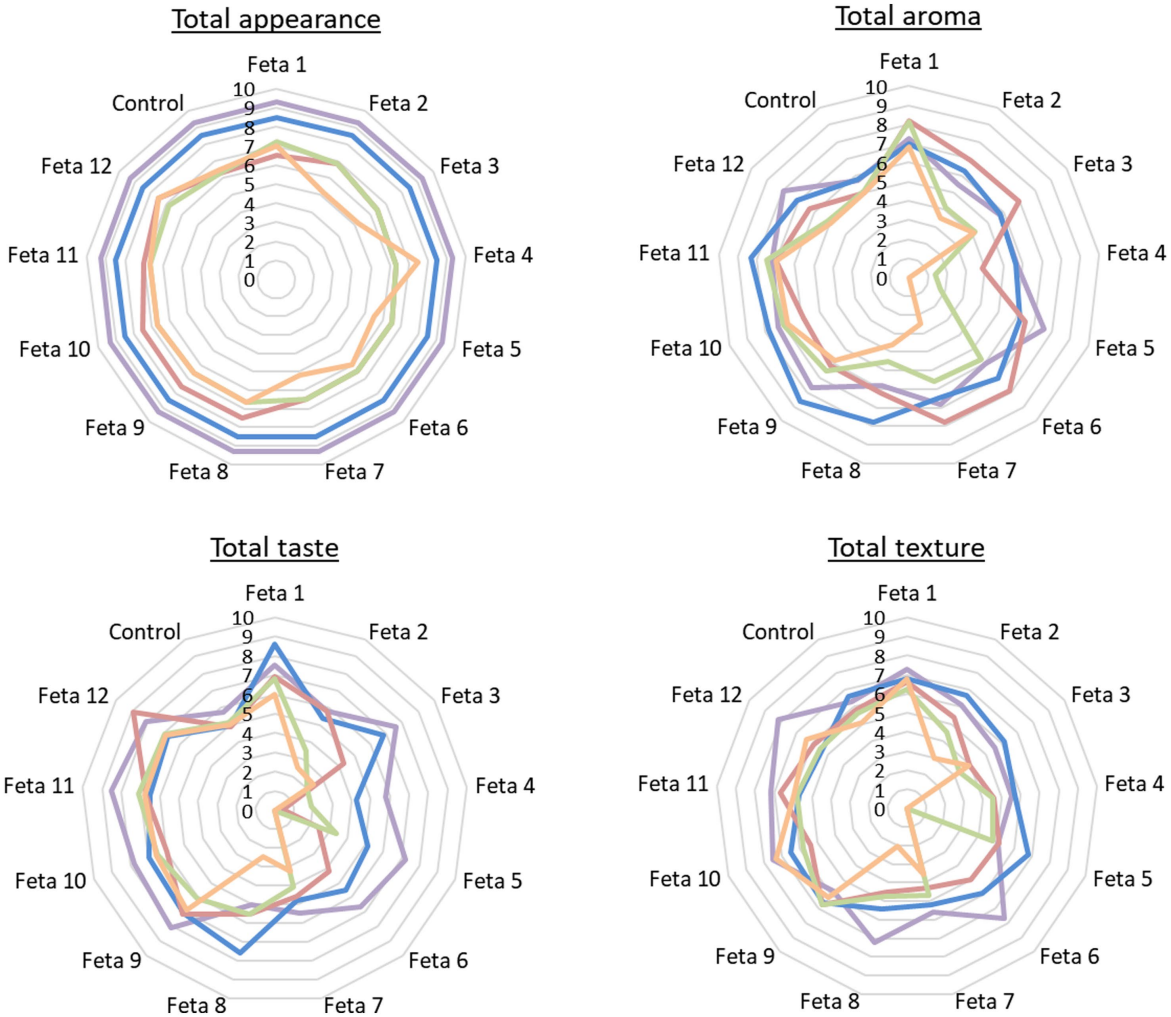
Sensory analysis of all Feta cheese trials on 60^th^ day (■60d), 90^th^ day (■90d), 120^th^ day (■120d), 150^th^ day (■150d) and 180^th^ day (■180d) after cheese production regarding total appearance, total aroma, total taste and total texture.

**Figure 4:**
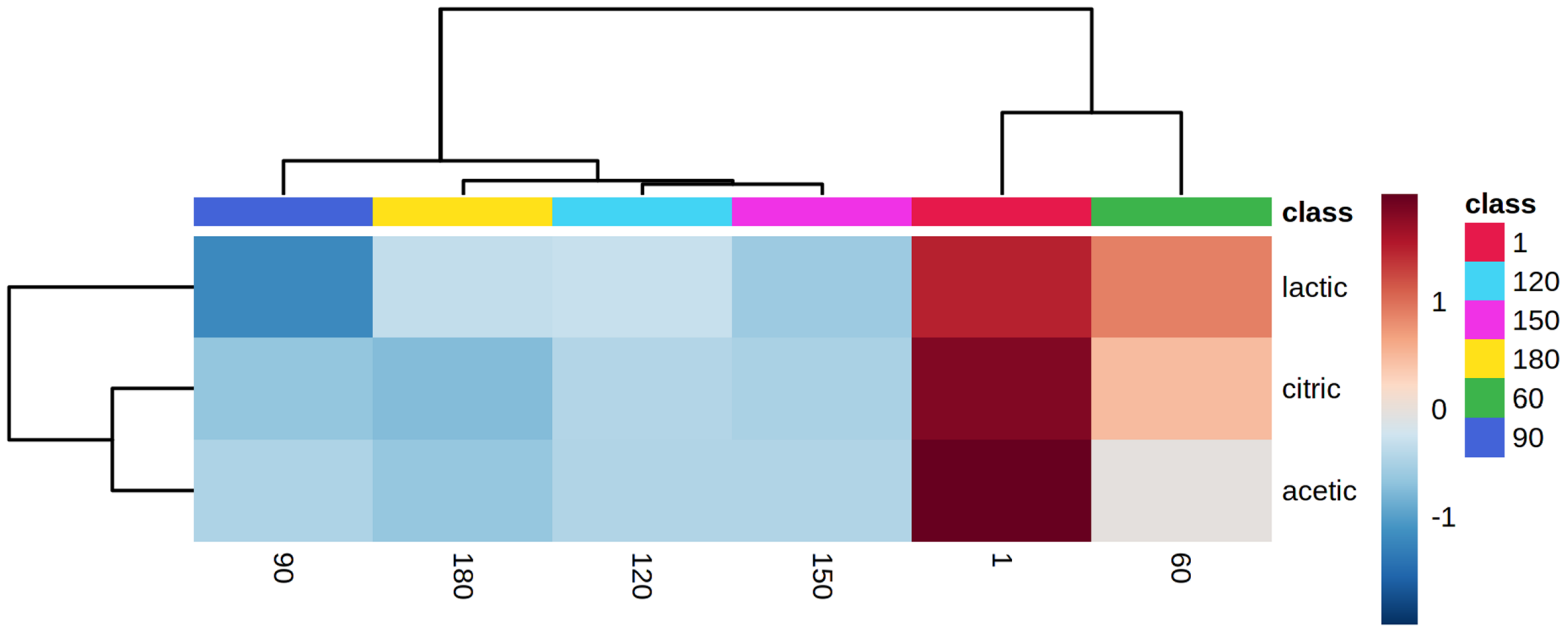
Hierarchical clustering result shown as a heatmap of organic acids associated with the different days (ripening and storage) of the 6 Feta cheese trials (1, 9, 10, 11, 12 and the control). Ward-linkage clustering was based on the Euclidean correlation coefficients of the identified organic acids in the different cheese trials. The color scale represents the scaled abundance of each variable, with red indicating high abundance and blue indicating low abundance.

During storage at 4°C, the pathogen was not detected after enrichment in Feta L12 trials (inoculated with acid and non-acid *Listeria* strains), as reported above, whereas in corresponding control trials, the pathogen was not detected after enrichment from the 90th day (storage) onwards.

The initial population of mesophilic lactobacilli and lactococci in Feta L12 and the control samples (day 1) inoculated with *Listeria* was 8.5–9.2 log CFU/g, in both batches. The results showed that both populations gradually decreased during ripening and storage in both trials, a result that was also observed in the non-inoculated Feta trials. More specifically, the population of mesophilic lactobacilli was reduced by 1.2–1.5 log CFU/g by the end of storage, while the population of lactococci was reduced by 1.0–1.9 log CFU/g in all trials (data not shown). The TVC population was found to be 8.6–9.0 log CFU/g, in both batches at day 1, while during ripening and storage the population gradually decreased (Figures 2A,B). The other examined microbiota (i.e., *Enterobacteriaceae*, staphylococci, yeasts and molds) was found below the detection limit of the enumeration method (data not shown). The initial pH values of the cheeses were 4.6–4.7 on the 1^st^ day of production in all samples, while during ripening and storage the pH values of all samples showed a slight decrease and obtained pH values of 4.2–4.4 (data not shown).

To prevent the growth and spread of *Listeria monocytogenes* in cheese, it is important to implement good manufacturing practices and food safety measures (Kapetanakou et al., 2017). Additionally, natural antimicrobial ingredients such as bacteriocins or probiotics are becoming increasingly popular as a way to prevent the growth of *L. monocytogenes* in food products, including cheese (Reis et al., 2012). Up to date, many studies have confirmed the anti-listerial activity of selected autochthonous LAB strains in cheeses (Dal Bello et al., 2012; Coelho et al., 2014; Morandi et al., 2019; Ivanovic et al., 2021). The use of such strains can lead to an effective reduction of the pathogen below the limit imposed by Commission Regulation (EC) No. 1441/2007 (<2.0 log CFU/g). Papadopoulou et al. (2018) studied the effect of *Lpb. plantarum* T571 on the fate of *L. monocytogenes* strains during ripening and storage of Feta. Their results showed that the added strain inactivated the pathogen during ripening and in a shorter time compared to the control samples. Pisano et al. (2022) investigated the use of three *Lc. lactis* strains and two *Lpb. plantarum* strains in limiting the growth of *L. monocytogenes* on laboratory fresh cheeses. These researchers reported that the examined strains could be used as protective cultures since they reduced the pathogen population by 3–4 log CFU/g compared to the control. Finally, in the present study, it deemed necessary to study the fate of *L. monocytogenes* strains after acid adaptation due to the high acid concentrations present in the Feta matrix (pH 4.6). However, the results of this study demonstrated that during ripening, significant differences (*p* < 0.05) were found between the treatments in the survival of the pathogen, while during storage, no significant differences (*p* ≥ 0.05) were found. Similar results were found in an Italian-style soft cheese, in which the acid adaptation of *Listeria* cells provided the same population levels at the examined cheeses as with the non-acid adapted strains (Cataldo et al., 2007).

### 3.3. Physicochemical analysis

pH values decreased in all trials from a range of 4.6–4.8 after production to 4.2–4.6 at the end of ripening, while a post-acidification was observed during storage, reaching values of 4.1 to 4.4 depending on the trial (data not shown). In more detail, until the end of ripening, Feta 12 demonstrated a pH of *ca.* 4.6, Feta 2, 6 and 10 had pH 4.4, Feta 1 and 8 had pH 4.3 while Feta 3, 4, 5, 7, 9, 11 had a similar pH value to the control trial (*ca.* 4.5). Until the end of storage, a reduction in pH values was observed in all Feta trials. More specifically, Feta 4, 5 and 9 had a pH of ca 4.4, Feta 1, 2, 8 and 10 had a pH value of 4.2, while Feta 3, 6, 7, 11 and 12 had a similar pH value to the control trial (*ca.* 4.3). The results obtained by FoodScan in the food industry at the beginning and end of storage for the 13 Feta trials, revealed minor differentiations between the trials (Supplementary Table S1).

According to Abd El-Salam and Alichanidis (2004) pH of Feta cheese reaches 4.4–4.6 during ripening at 16–18°C and its moisture decreases to <56% before release to the market. According to Kourkoutas et al. (2006), Dimitrellou et al. (2014) and Papadopoulou et al. (2018), who studied Feta and WBC produced with the addition of adjunct LAB strains, a post-acidification is usually apparent with pH reaching values of 4.4 or lower, a result that was also observed in the current study.

### 3.4. Sensory evaluation

The results of the sensory evaluation of the 13 Feta trials (without the pathogen) are presented in Figure 3. All the results are presented in comparison to the control trial. In general, the nine selected LAB strains mixed in 12 different combinations produced cheese products with sensorial characteristics that differentiated them from the control.

From the evaluation of the total appearance, it was observed that all the Feta trials were considered acceptable until the end of storage. More specifically, until the 90^th^ day all Feta trials were evaluated with high scores (approximately 9/10). However, from 120^th^ up to the 180^th^ day, there was a variation in scores. Feta trials 1, 2, 7, 8, 9, 10, 11, and 12 received scores between 7 and 8/10, while Feta trials 3, 4, 5, and 6, along with the control, were rated with 6/10. At the end of storage, the Feta trials 4 and 12 were scored higher (8/10) than the control (7/10) and the Feta trials 2, 3, 5, 6, and 7 were scored lower (approximately 5/10) than the control. This decline in scores was attributed to the presence of irregular holes and cracks, as opposed to the small mechanical openings found in the control (p ≥ 0.05).

The evaluation of the overall aroma indicated that all the Feta trials were acceptable until the 90^th^ day, with the majority of them receiving higher scores (ranging from 7 to 8/10) compared to the control (rated at 6/10). On 120^th^ day, the Feta trials 1, 2, 3, 5, 6, 7, 8, 9, 10, 11, and 12, received higher scores (ranging from 6 to 8 out of 10) compared to the control (rated at 5/10), while the Feta trial 4 was considered unacceptable (score 4/10). On 150^th^ day, Feta trials 1, 6, 7, 9, 10 and 11 were evaluated with scores of up to 7/10. Feta trials 8, 12 and control received scores of 5/10, while the Feta trials 2, 3, 4 and 5 were considered unacceptable with scores ranging from 1 to 4/10. At the end of storage period (180^th^ day), Feta trials 1, 9, 10 and 11 were evaluated with scores of 6–7/10. Feta trials 12 and control received scores of 5/10, while the remaining trials were considered unacceptable (*p* < 0.05). The presence of the intense acid aroma in several trials during the later stages of storage had significantly impacted their overall aroma scores.

Regarding the evaluation of the overall taste, the majority of Feta trials were scored higher than the control until the 120^th^ day, while later the Feta trials showed variability in their scores. In more detail, at the end of ripening the Feta trials 1, 3, 5, 6, 9, 10, 11, and 12 received scores from 7 to 9/10, Feta trials 2, 4 and control 6/10, while the Feta trials 7 and 8 receive scores 5/10. On 90^th^ day Feta trials 1, 3, 6, 7, 8, 9, 10, 11, and 12, received scores ranging from 7 to 9/10, Feta trials 2, 5, 7 and control rated at 5/10, while the Feta trial 4 was consider unacceptable (score 4/10). On 120^th^ day, Feta trials 1, 2, 8, 7, 9, 10, 11 and 12 were evaluated with scores from 7 to 9/10. Feta trials 7 and control received scores of 5/10, while the Feta trials 3, 4, 5 and 6 were considered unacceptable with scores ranging from 0 to 4/10. On 150^th^ day Feta trials 1, 9, 10 and 11 were evaluated with higher scores (6–7/10) compared to the control (5/10), while the others scored lower than the control rated from 0 to 4/10. At the end of storage (180^th^ day), Feta trials 1, 9, 10, 11 and 12 were evaluated with higher scores (from 6 to 7/10) compared to the control (5/10), while the remaining trials were considered unacceptable (scores from 0 to 3/10) (p < 0.05). The Feta trials that were found unacceptable displayed intense increase of characteristics “bitter” and “acid.”

Finally, regarding the overall texture, trials 3, 4, 7 and 8 were considered unacceptable from the 120^th^ day, while the Feta trials 2, 5, and 6 were considered unacceptable from the 150^th^ day and beyond. Τheir exclusion was attributed to their bad consistency and granular texture (p < 0.05). The trials with high scores (from 7 to 8/10) in the feature “overall texture” were Feta 1, 9, 10, 11, 12. From the above results, it is apparent that the Feta trials 1, 9, 10, 11 and 12 were acceptable until the end of the storage and were graded with better scores in the evaluation of all sensorial characteristics compared to the control. Most of these trials included cheeses produced with mixed cultures (i.e., Feta 9, 10, 11 and 12), while Feta 1 was the only cheese trial that was produced with the monoculture of *Lcb. paracasei* SRX10. A detailed description of the selected Feta trials sensory characteristics after ripening and at the end of storage is presented in Supplementary Figure S3.

With regards to sensory discrimination, the results from hierarchical cluster analysis (Supplementary Figure S4) showed that the cheeses were classified into three (3) main clusters. One cluster contained all cheese trials from early storage (60 and 90 days after production), while the other two (2) clusters contained samples from middle (120 and 150 day) and final (180 day) storage days. Trials from 180 days were mostly classified in one cluster except for 4 trials, while cheese trials from 120 and 150 days were rather mixed. No discrimination between different cheese trials were found, yet the cheeses were mostly classified between early and middle/final storage times.

Feta has pleasant organoleptic properties that has worldwide acceptance. Some of the organoleptic characteristics of this cheese are the salty and slightly acidic taste, smoothy, creamy and firm texture, which make the cheese easy to cut into slices (Sarantinopoulos et al., 2002). As regards texture, no gas holes should be present, but irregular small mechanical openings are desirable (Anifantakis, 1991; Abd El-Salam et al., 1993). In this study, the presence of uneven holes and cracks in many Feta trials can be attributed to the heterofermentative nature of the added LAB strains (i.e., *Ln. mesenteroides* FRX4, FMX3 and *Lvb.* brevis SRX20), which are able to produce gas, a property that was previously studied in Kamarinou et al. (2022). Several LAB strains have been used as adjunct cultures in the manufacture of different types of cheese and their addition may affect the organoleptic characteristics of the products thus, a sensory assessment is crucial. Ahmed et al. (2021) used *Lcb. casei* LBC, *B. bifidum BB* and *Lc. lactis* subsp. *lactis* LL as adjunct cultures in the manufacture of low-fat Feta-type cheese. Their results showed that the cheese with the added strains presented a good appearance and color throughout storage (Ahmed et al., 2021). Mantzourani et al. (2018) demonstrated that the addition of *Lcb. paracasei* SP3 in a Feta-type cheese, improved the organoleptic characteristics of the product. In the study of Papadopoulou et al. (2018), it was shown that the addition of *Lpb. plantarum* T571 as an adjunct culture in a pilot Feta cheese production influenced positively the overall texture of the cheese during storage at 4°C. In contrast, the study of Yousefi et al. (2021) showed that no significant differences were found between the sensory characteristics of ultrafiltered Feta-type cheese containing the adjunct culture *Lvb. brevis* KX572376 and the control. Many of the aforementioned studies indicated that selected LAB strains can lead to the production of cheeses with desirable organoleptic characteristics, a result that is in accordance with the current study at selected Feta trials (1, 9, 10, 11 and 12). As regards the differentiation between the mono and mixed cultures, it was observed that the addition of *Ln. mesenteroides* FMX3 as a monoculture (Feta 5) affected the attributes of Feta cheese negatively, while the addition of *Ln. mesenteroides* FMX3 as a mixed culture (Feta 12) improved the organoleptic characteristics of the cheese. Due to the heterofermentative metabolism of this genus, Server-Busson et al. (1999) suggested that *Leuconostoc* isolates should be used in combination with acid-producing lactococci, as starters or adjuncts, and not as monocultures. Finally, it should be highlighted that the use of *Lcb. paracasei* SRX10 as an adjunct monoculture in the present study, produced Feta cheese with high scores (>6) in all of the examined organoleptic characteristics (appearance, aroma, taste and texture), This result was also confirmed by other studies, where it was shown that the addition of several *Lcb. paracasei* strains as adjunct cultures enhanced the flavor formation and increased the overall quality of cheeses, i.e., in a short-ripened Italian semi-hard cheese (Bancalari et al., 2020), in a Feta-type cheese (Bintsis and Robinson, 2004) and in a WBC (Terpou et al., 2018b).

### 3.5. HPLC analysis of organic acids

Six (6) cheese trials (Feta 1, 9, 10, 11, 12 and control) were selected after the sensory analysis (scores above 5 in the hedonic scale) to undergo HPLC analysis. The analysis of the chromatograms from HPLC resulted in the discrimination of three organic acids, i.e., citric acid, lactic acid and acetic acid. The other examined acids, i.e., tartaric, succinic, formic, and propionic, were not detected in any of the samples. In details, citric acid concentrations were estimated to be between 2.14–3.33 mg/g after cheese production (day 1) and reduced during storage in trials 1, 9, 10 and 12 to 0.1–0.7 mg/g, except in trial 9, where the acid was not detected during storage (*p* < 0.05). Lactic acid concentration displayed the highest levels in all cheese trials and was estimated between 16.3–22.0 mg/g after cheese production, depending on the different trials (p < 0.05). During storage, a small decline was observed in all trials, where at the end of storage, the concentration of lactic acid was estimated between 9.94–14.82 mg/g. Acetic acid concentration was found in traces throughout ripening and storage (*p* ≥ 0.05). After ANOVA, no significant difference was found between the concentrations of organic acids in the six Feta trials (p ≥ 0.05). As regards storage discrimination, the results from PLS-DA showed that citric and lactic acids were highly correlated (VIP scores>1) with fresh samples (day 1) (data not shown). The obtained dendrogram (Supplementary Figure S5) showed that the samples were classified into two main clusters. One cluster contained samples from days 1 and 60, and the second cluster contained samples from days 90, 120, 150 and 180 days. Figure 4 represents the heatmap obtained from the combination of storage days and the organic acid concentration. It can be observed that all acids were highly correlated with the ripening period rather than the storage period. To conclude, it was observed that the changes were more evident due to the different storage days than the different adjunct mono and mixed cultures used in the six examined trials, a trend that was also evident for the sensory changes in the 13 cheese trials, as described previously.

According to previous studies, the organic acids that are present in Feta and Feta-type cheeses during ripening are mainly citric, acetic and lactic acids (Papadakis and Polychroniadou, 2005; Manolaki et al., 2006). Lactic acid, the major product of sugar fermentation by LAB, is the most abundant organic acid present in cheeses, where its concentration can vary from 1.94 to 17.4 mg/g in aged cheeses (Kaminarides et al., 2007). Among the other detected acids, citric acid is usually lost in the whey during cheese production, however, the retained citric acid may be metabolized to volatile compounds (i.e., acetic acid, diacetyl, etc.) by some LAB, such as *Lc. lactis* subsp. *diacetylactis* and *Leuconostoc* spp. (Izco et al., 2002; Kaminarides et al., 2007; Jo et al., 2018). Consequently, acetic acid can contribute to cheese flavor (Jo et al., 2018). As shown above, Feta trials showed a high concentration of lactic acid, in contrast to the citric and acetic acids that were detected at lower levels. Comparable results were observed by Akalin et al. (2002), who studied the organic acids in WBC and demonstrated that lactic acid accounted for about 95% of the total organic acid content. In addition, Papadopoulou et al. (2022) investigated the changes in organic acids during storage of a semi-hard bovine sliced cheese supplement with a cocktail of 3 LAB strains and their results showed that lactic acid concentration displayed the highest levels. In accordance with the above, in the current study, it was also observed that the concentration of citric and lactic acids was found to be higher after cheese production (day 1) and declined during storage. Finally, Sabbagh et al. (2010) observed that during the ripening of Iranian low-fat white cheese, the concentrations of lactic and acetic acids decreased, a result that was in accordance with the current study.

### 3.6. RAPD-PCR for monitoring LAB survival and strain differentiation

**Figure 5:**
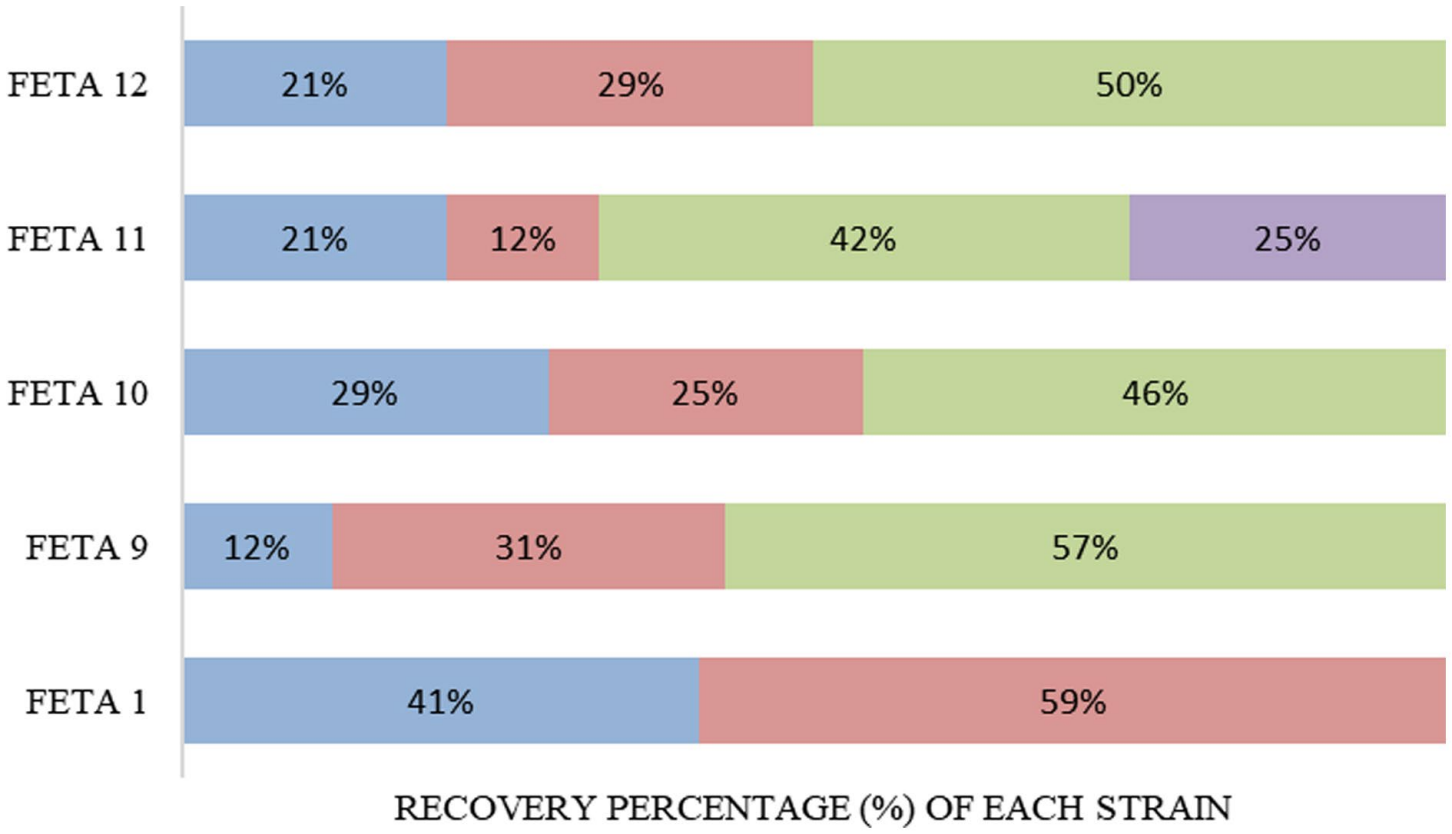
Recovery rate of the adjunct functional LAB strains at 180 days (end of storage). Commercial Starter culture **(■), Feta 1:** *Lcb. casei* SRX10 **(■), Feta 9:** *Ln. mesenteroides* FRX4 **(■),** *Lc. lactis* SMX16 **(■), Feta 10:** *Lcb. paracasei* SRX10 **(■),** *Lpb. plantarum* FB1 **(■), Feta 11:** *Ln. mesenteroides* FMX11 **(■),** *Lvb. brevis* SRX20 **(■),** *Lpb. plantarum* FRX20 **(■), Feta 12:** *Ln. mesenteroides* FMX3 **(■),** *Lc. lactis* SMX2 **(■)**.

The population of LAB was maintained at over 6.0 log CFU/g until the end of storage in the 5 selected cheese trials (Feta 1, 9, 10, 11 and 12; received scores over 5 in the hedonic scale), as shown previously in Figure 1. So, it was crucial to verify the presence and survival of the added multi-functional LAB strains in a harsh environment, such as the Feta cheese. The observation of RAPD-PCR profiles revealed that the total of the recovered isolates in Feta cheese trials belonged to the adjunct and CS cultures, but in different percentages, depending on the different Feta trials (Figure 5). In brief, in Feta 1, *Lcb. paracasei* SRX10, which was used as an adjunct monoculture, survived well until the end of storage and showed a high recovery percentage (59%) compared to the CS culture (41%). Similarly, in Feta 9, the adjunct mixed culture presented a high recovery percentage, i.e., 31% for *Ln. mesenteroides* FRX4 and 57% for *Lc. lactis* SMX16. In Feta 10, the strain *Lpb. plantarum* FB1 exhibited a higher recovery percentage (46%) than the strain *Lcb. paracasei* SRX10 and the CS culture, which shared similar recovery percentages (25 and 29%, respectively). In Feta 11, the strain distribution was *Lvb. brevis* SRX20 42%, *Lpb. plantarum* FRX20 25%, *Ln. mesenteroides* FMX11 12% and CS culture 21%. Finally, in Feta 12, the strain *Lc. lactis* SMX2 was recovered in a high percentage (50%), followed by *Ln. mesenteroides* FMX3 (29%) and the CS culture (21%).

Feta cheese is a dynamic ecosystem, and its microbiota is constantly changing during ripening and storage, where salting (*ca.* 7%) and/or the low pH (4.4–4.6) can affect the survival of selected species or strains. From the RAPD analysis results, it was evident that the autochthonous added strains dominated the LAB community in all the examined Feta trials. It is known that lactobacilli can grow and survive in fermented food products with low pH values (3.7–4.3) (Tripathi and Giri, 2014), where *Leuconostoc* spp. as NSLAB are naturally occurring in various stages during cheese ripening and storage (Seixas et al., 2018). Angelopoulou et al. (2017) and Papadopoulou et al. (2018) have also confirmed the survival of the adjunct cultures used for Feta or Feta-type production during ripening and/or storage of the cheeses by using molecular tools. However, limited research is available for Feta cheese produced with a cocktail of autochthonous LAB strains as well as for their survival in the final product. The aforementioned studies have dealt with the addition of one autochthonous strain for cheese production, whereas in the present study, multiple combinations were made. For that reason, it was essential to study the prevailing microbiota to confirm the survival of the added LAB strains by using a molecular tool such as RAPD-PCR.

## 4. Conclusion

The findings of the present study revealed that the incorporation of many of the examined multifunctional LAB strains led to products with desirable sensory properties (salty and slightly acidic taste, smoothy, creamy and firm texture with small mechanical openings and high scores in the “overall taste” and “overall aroma” attributes), without altering but enhancing the traditional character of Feta cheese. Furthermore, as regards the safety of the novel products, the 2 strains with anti-listerial activity *in vitro* (*Ln. mesenteroides* FMX3 and *Lc. lactis* SMX2) provided encouraging results, since the pathogen was eliminated in a shorter time in contrast to the CS culture and before the product release in the market (i.e., 60 days). In addition, many of the added strains were able to survive and thrive in the harsh Feta environment in adequate populations to consider the product a functional food.

Although further research is needed, the results of the present study are promising for the production of new dairy products with enhanced quality, safety and high added value, by using selected LAB strains. In this respect, *Lcb. paracasei* SRX10 and the anti-listerial *Ln. mesenteroides* FMX3 and *Lc. lactis* SMX2 strains are promising adjunct candidates to develop functional Feta cheese with distinctive sensory character (SRX10) and enhanced safety (FMX3 and SMX2). In addition, bioinformatic analysis of these indigenous multi-functional strains may reveal valuable information about specific genes with safety aspects, technological properties and/or functional properties. Furthermore, the use of molecular tools for the investigation of the relationship between the cheese environment and the bacterial functionality, will be a desired aspect, leading to fermented products with exceptional properties.

## Data availability statement

The raw data supporting the conclusions of this article will be made available by the authors, without undue reservation.

## Author contributions

CK: Conceptualization, Data curation, Formal analysis, Investigation, Methodology, Software, Visualization, Writing – original draft. OP: Conceptualization, Data curation, Formal analysis, Investigation, Methodology, Software, Supervision, Visualization, Writing – review & editing. AD: Formal analysis, Investigation, Methodology, Supervision, Visualization, Writing – review & editing. CT: Conceptualization, Visualization, Writing – review & editing. AG: Methodology, Supervision, Visualization, Writing – review & editing. NC: Conceptualization, Funding acquisition, Investigation, Methodology, Project administration, Supervision, Visualization, Writing – review & editing. AA: Conceptualization, Data curation, Formal analysis, Funding acquisition, Investigation, Methodology, Project administration, Software, Supervision, Visualization, Writing – review & editing.

## References

[ref1] Abd El-Salam M. H. Alichanidis E. (2004). “Cheese varieties ripened in brine” in Cheese: Chemistry, physics and microbiology. Eds. Fox P. F. McSweeney P. L. H. Cogan T. M. Guinee T. P.. (Academic Press), 227–249.

[ref2] Abd El-Salam M. H. Alichanidis E. Zerfiridis G. K. (1993). “Domiati and feta type cheeses” in Cheese: Chemistry, physics and microbiology. Ed. Fox P. F.. (Boston, MA: Springer), 301–335.

[ref3] Ahmed M. E. Rathnakumar K. Awasti N. Elfaruk M. S. Hammam A. R. (2021). Influence of probiotic adjunct cultures on the characteristics of low-fat feta cheese. Food Sci. Nutr. 9, 1512–1520. doi: 10.1002/fsn3.2121, PMID: 33747465 PMC7958540

[ref4] Akalin A. S. Gönç S. Akbaş Y. (2002). Variation in organic acids content during ripening of pickled white cheese. J. Dairy Sci. 85, 1670–1676. doi: 10.3168/jds.S0022-0302(02)74239-2, PMID: 12201516

[ref5] Angelopoulou A. Alexandraki V. Georgalaki M. Anastasiou R. Manolopoulou E. Tsakalidou E.. (2017). Production of probiotic feta cheese using *Propionibacterium freudenreichii* subsp. shermanii as adjunct. Int. Dairy J. 66, 135–139. doi: 10.1016/j.idairyj.2016.11.011

[ref6] Anifantakis E. M. (1991). Traditional feta cheese. Feta and related cheeses, eds. Robinson R. K. Tamime A. Y.. A volume in Woodhead Publishing Series in Food Science, Technology and Nutrition. 49–69. doi: 10.1007/978-94-011-3824-6_2

[ref7] Argyri A. A. Doulgeraki A. I. Blana V. A. Panagou E. Z. Nychas G. J. E. (2011). Potential of a simple HPLC-based approach for the identification of the spoilage status of minced beef stored at various temperatures and packaging systems. Int. J. Food Microbiol. 150, 25–33. doi: 10.1016/j.ijfoodmicro.2011.07.010, PMID: 21835483

[ref8] Arslan S. Özdemir F. (2008). Prevalence and antimicrobial resistance of Listeria spp. in homemade white cheese. Food Control 19, 360–363. doi: 10.1016/j.foodcont.2007.04.009

[ref9] Bancalari E. Montanari C. Levante A. Alinovi M. Neviani E. Gardini F.. (2020). Lactobacillus paracasei 4341 as adjunct culture to enhance flavor in short ripened Caciotta-type cheese. Food Res. Int. 135:109284. doi: 10.1016/j.foodres.2020.109284, PMID: 32527479

[ref10] Bettera L. Levante A. Bancalari E. Bottari B. Gatti M. (2022). Lactic acid bacteria in cow raw milk for cheese production: which and how many? Front. Microbiol. 13:1092224. doi: 10.3389/fmicb.2022.1092224 36713157 PMC9878191

[ref11] Bintsis T. Papademas P. (2002). Microbiological quality of white-brined cheeses: A review. Int. J. Dairy Technol. 55, 113–120. doi: 10.1046/j.1471-0307.2002.00054.x

[ref12] Bintsis T. Robinson R. K. (2004). A study of the effects of adjunct cultures on the aroma compounds of feta-type cheese. Food Chem. 88, 435–441. doi: 10.1016/j.foodchem.2004.01.057

[ref13] Cadavez V. A. Campagnollo F. B. Silva R. A. Duffner C. M. Schaffner D. W. Sant’Ana A. S.. (2019). A comparison of dynamic tertiary and competition models for describing the fate of *Listeria monocytogenes* in Minas fresh cheese during refrigerated storage. Food Microbiol. 79, 48–60. doi: 10.1016/j.fm.2018.11.004, PMID: 30621875

[ref14] Campagnollo F. B. Gonzales-Barron U. Pilão Cadavez V. A. Sant’Ana A. S. Schaffner D. W. (2018). Quantitative risk assessment of *Listeria monocytogenes* in traditional Minas cheeses: the cases of artisanal semi-hard and fresh soft cheeses. Food Control 92, 370–379. doi: 10.1016/j.foodcont.2018.05.019

[ref15] Cataldo G. Conte M. P. Chiarini F. Seganti L. Ammendolia M. G. Superti F.. (2007). Acid adaptation and survival of *Listeria monocytogenes* in Italian-style soft cheeses. J. Appl. Microbiol. 103, 185–193. doi: 10.1111/j.1365-2672.2006.03218.x, PMID: 17584464

[ref16] Cocolin L. Diez A. Urso R. Rantsiou K. Comi G. Bergmaier I.. (2007). Optimization of conditions for profiling bacterial populations in food by culture-independent methods. Int. J. Food Microbiol. 120, 100–109. doi: 10.1016/j.ijfoodmicro.2007.06.015, PMID: 17604862

[ref17] Coelho M. C. Silva C. C. G. Ribeiro S. C. Dapkevicius M. L. N. E. Rosa H. J. D. (2014). Control of *Listeria monocytogenes* in fresh cheese using protective lactic acid bacteria. Int. J. Food Microbiol. 191, 53–59. doi: 10.1016/j.ijfoodmicro.2014.08.029, PMID: 25222327

[ref18] Dal Bello B. Cocolin L. Zeppa G. Field D. Cotter P. D. Hill C. (2012). Technological characterization of bacteriocin producing *Lactococcus lactis* strains employed to control *Listeria monocytogenes* in cottage cheese. Int. J. Food Microbiol. 153, 58–65. doi: 10.1016/j.ijfoodmicro.2011.10.016, PMID: 22104121

[ref19] de Souza J. V. Dias F. S. (2017). Protective, technological, and functional properties of select autochthonous lactic acid bacteria from goat dairy products. Curr. Opin. Food Sci. 13, 1–9. doi: 10.1016/j.cofs.2017.01.003

[ref20] Dimitrellou D. Kandylis P. Sidira M. Koutinas A. A. Kourkoutas Y. (2014). Free and immobilized *Lactobacillus casei* ATCC 393 on whey protein as starter cultures for probiotic feta-type cheese production. J. Dairy Sci. 97, 4675–4685. doi: 10.3168/jds.2013-7597, PMID: 24931523

[ref21] Escobar-Zepeda A. Sanchez-Flores A. Baruch M. Q. (2016). Metagenomic analysis of a Mexican ripened cheese reveals a unique complex microbiota. Food Microbiol. 57, 116–127. doi: 10.1016/j.fm.2016.02.004, PMID: 27052710

[ref22] Fox P. F. Guinee T. P. Cogan T. M. McSweeney P. L. (2017). “Microbiology of cheese ripening” in Fundamentals of cheese science. Eds. Fox P. F. Guinee T. P. Cogan T. M. McSweeney P. L. H.. (Boston, MA: Springer), 333–390.

[ref23] Gaglio R. Todaro M. Settanni L. (2021). Improvement of raw milk cheese hygiene through the selection of starter and non-starter lactic acid bacteria: the successful case of PDO pecorino Siciliano cheese. Int. J. Environ. Res. Public Health 18:1834. doi: 10.3390/ijerph18041834, PMID: 33668630 PMC7917940

[ref24] Giraffa G. Rossetti L. Neviani E. (2000). An evaluation of Chelex-based DNA purification protocols for the typing of lactic acid Bacteria. J. Microbiol. Methods 42, 175–184. doi: 10.1016/S0167-7012(00)00172-X, PMID: 11018274

[ref25] González L. Zarate V. (2012). Influence of an autochthonous starter culture and a commercial starter on the characteristics of Tenerife pasteurised goats’ milk cheese. Int. J. Dairy Technol. 65, 542–547. doi: 10.1111/j.1471-0307.2012.00862.x

[ref26] Hayaloglu A. A. (2016). Cheese: microbiology of cheese. Reference module food sci 1, 1–11. doi: 10.1016/B978-0-08-100596-5.00675-2

[ref27] International Organizational for Standardization. (2017a). Microbiology of food chain - horizontal method for the detection, enumeration and serotyping of Salmonella - part 1: detection of Salmonella spp, International Organization for Standardization, Geneva.

[ref28] International Organizational for Standardization. (2017b). Microbiology of the food chain - horizontal method for the detection and enumeration of Listeria monocytogenes and of Listeria spp. - part 1: Detection method. International Organization for Standardization, Geneva.

[ref29] Ivanovic M. Mirkovic N. Mirkovic M. Miocinovic J. Radulovic A. Solevic Knudsen T.. (2021). Autochthonous *Enterococcus durans* PFMI565 and *Lactococcus lactis* subsp. lactis BGBU1–4 in bio-control of *Listeria monocytogenes* in ultrafiltered cheese. Foods 10:1448. doi: 10.3390/foods10071448, PMID: 34206521 PMC8304694

[ref30] Izco J. M. Tormo M. Jiménez-Flores R. (2002). Rapid simultaneous determination of organic acids, free amino acids, and lactose in cheese by capillary electrophoresis. J. Dairy Sci. 85, 2122–2129. doi: 10.3168/jds.S0022-0302(02)74290-2, PMID: 12362443

[ref31] Jo Y. Benoist D. M. Ameerally A. Drake M. A. (2018). Sensory and chemical properties of gouda cheese. J. Dairy Sci. 101, 1967–1989. doi: 10.3168/jds.2017-13637 29274971

[ref32] Kamarinou C. S. Papadopoulou O. S. Doulgeraki A. I. Tassou C. C. Galanis A. Chorianopoulos N. G.. (2022). Mapping the key technological and functional characteristics of indigenous lactic acid Bacteria isolated from Greek traditional dairy products. Microorganisms 10:246. doi: 10.3390/microorganisms10020246, PMID: 35208701 PMC8875946

[ref33] Kaminarides S. Stamou P. Massouras T. (2007). Changes of organic acids, volatile aroma compounds and sensory characteristics of halloumi cheese kept in brine. Food Chem. 100, 219–225. doi: 10.1016/j.foodchem.2005.09.039

[ref34] Kapetanakou A. E. Gkerekou M. A. Vitzilaiou E. S. Skandamis P. N. (2017). Assessing the capacity of growth, survival, and acid adaptive response of *Listeria monocytogenes* during storage of various cheeses and subsequent simulated gastric digestion. Int. J. Food Microbiol. 246, 50–63. doi: 10.1016/j.ijfoodmicro.2017.01.015, PMID: 28189900

[ref35] Kourkoutas Y. Bosnea L. Taboukos S. Baras C. Lambrou D. Kanellaki M. (2006). Probiotic cheese production using *Lactobacillus casei* cells immobilized on fruit pieces. J. Dairy Sci. 89, 1439–1451. doi: 10.3168/jds.S0022-0302(06)72212-3, PMID: 16606715

[ref36] Kousta M. Mataragas M. Skandamis P. Drosinos E. H. (2010). Prevalence and sources of cheese contamination with pathogens at farm and processing levels. Food Control 21, 805–815. doi: 10.1016/j.foodcont.2009.11.015

[ref37] Leroy F. De Vuyst L. (2004). Lactic acid bacteria as functional starter cultures for the food fermentation industry. Trends Food Sci. Technol. 15, 67–78. doi: 10.1016/j.tifs.2003.09.004

[ref38] Manolaki P. Katsiari M. C. Alichanidis E. (2006). Effect of a commercial adjunct culture on organic acid contents of low-fat feta-type cheese. Food Chem. 98, 658–663. doi: 10.1016/j.foodchem.2005.06.031

[ref39] Mantzourani I. Terpou A. Alexopoulos A. Chondrou P. Galanis A. Bekatorou A.. (2018). Application of a novel potential probiotic *Lactobacillus paracasei* strain isolated from kefir grains in the production of feta-type cheese. Microorganisms 6:121. doi: 10.3390/microorganisms6040121, PMID: 30501107 PMC6313735

[ref41] Michaelidou A. Katsiari M. C. Kondyli E. Voutsinas L. P. Alichanidis E. (2003). Effect of a commercial adjunct culture on proteolysis in low-fat feta-type cheese. Int. Dairy J. 13, 179–189. doi: 10.1016/S0958-6946(02)00148-6

[ref42] Michailidou S. Pavlou E. Pasentsis K. Rhoades J. Likotrafiti E. Argiriou A. (2021). Microbial profiles of Greek PDO cheeses assessed with amplicon metabarcoding. Food Microbiol. 99:103836. doi: 10.1016/j.fm.2021.103836, PMID: 34119120

[ref43] Morandi S. Silvetti T. Battelli G. Brasca M. (2019). Can lactic acid bacteria be an efficient tool for controlling *Listeria monocytogenes* contamination on cheese surface? The case of Gorgonzola cheese. Food Control 96, 499–507. doi: 10.1016/j.foodcont.2018.10.012

[ref44] Osaili T. M. Al-Nabulsi A. A. Taha M. H. Al-Holy M. A. Alaboudi A. R. Al-Rousan W. M.. (2012). Occurrence and antimicrobial susceptibility of *Listeria monocytogenes* isolated from brined white cheese in Jordan. J. Food Sci. 77, M528–M532. doi: 10.1111/j.1750-3841.2012.02877.x, PMID: 22897495

[ref45] Papadakis P. Konteles S. Batrinou A. Ouzounis S. Tsironi T. Halvatsiotis P.. (2021). Characterization of bacterial microbiota of PDO feta cheese by 16S metagenomic analysis. Microorganisms 9:2377. doi: 10.3390/microorganisms9112377, PMID: 34835502 PMC8625534

[ref46] Papadakis E. N. Polychroniadou A. (2005). Application of a microwave-assisted extraction method for the extraction of organic acids from Greek cheeses and sheep milk yoghurt and subsequent analysis by ion-exclusion liquid chromatography. Int. Dairy J. 15, 165–172. doi: 10.1016/j.idairyj.2004.06.006

[ref47] Papadimitriou K. Anastasiou R. Georgalaki M. Bounenni R. Paximadaki A. Charmpi C.. (2022). Comparison of the microbiome of artisanal homemade and industrial feta cheese through amplicon sequencing and shotgun metagenomics. Microorganisms 10:1073. doi: 10.3390/microorganisms10051073, PMID: 35630516 PMC9146562

[ref48] Papadopoulou O. S. Argyri A. A. Bikouli V. C. Lambrinea E. Chorianopoulos N. (2022). Evaluating the quality of cheese slices packaged with Na-alginate edible films supplemented with functional lactic acid Bacteria cultures after high-pressure processing. Foods 11:2855. doi: 10.3390/foods11182855, PMID: 36140989 PMC9498243

[ref49] Papadopoulou O. S. Argyri A. A. Varzakis E. E. Tassou C. C. Chorianopoulos N. G. (2018). Greek functional feta cheese: enhancing quality and safety using a *Lactobacillus plantarum* strain with probiotic potential. Food Microbiol. 74, 21–33. doi: 10.1016/j.fm.2018.02.005, PMID: 29706334

[ref50] Papetti P. Carelli A. (2013). Composition and sensory analysis for quality evaluation of a typical Italian cheese: influence of ripening period. Czech J. Food Sci. 31, 438–444. doi: 10.17221/447/2012-CJFS

[ref51] Pisano M. B. Fadda M. E. Viale S. Deplano M. Mereu F. Blažić M.. (2022). Inhibitory effect of Lactiplantibacillus plantarum and *Lactococcus lactis* autochtonous strains against *Listeria monocytogenes* in a laboratory cheese model. Foods 11:715. doi: 10.3390/foods11050715, PMID: 35267348 PMC8909851

[ref52] Pisano M. B. Rosa A. Putzu D. Cesare Marincola F. Mossa V. Viale S.. (2020). Influence of autochthonous putative probiotic cultures on microbiota, lipid components and metabolome of Caciotta cheese. Front. Microbiol. 11:583745. doi: 10.3389/fmicb.2020.583745 33193226 PMC7609418

[ref53] Prezzi L. E. Lee S. H. Nunes V. M. Corassin C. H. Pimentel T. C. Rocha R. S.. (2020). Effect of *Lactobacillus rhamnosus* on growth of Listeria monocytogenes and *Staphylococcus aureus* in a probiotic Minas Frescal cheese. Food Microbiol. 92:103557. doi: 10.1016/j.fm.2020.103557, PMID: 32950151

[ref54] Psoni L. Tzanetakis N. Litopoulou-Tzanetaki E. (2006). Characteristics of Batzos cheese made from raw, pasteurized and/or pasteurized standardized goat milk and a native culture. Food Control 17, 533–539. doi: 10.1016/j.foodcont.2005.03.001

[ref55] RASFF (2022). (The rapid alert system for food and feed). Available at: https://webgate.ec.europa.eu/rasff-window/screen/notification/582129

[ref56] Regulation (EC) No 1441/2007 of the European Parliament and of the Council of 5 Amending regulation (EC) no 2073/2005 on microbiological criteria for foodstuffs; European Parliament: Brussels, Belgium, (2007)

[ref57] Regulation (EC) no 1829/2002 of the European Parliament and of the Council of 14 October 2002 Amending the annex to regulation (EC) no 1107/96 with regard to the name ‘feta; European Parliament: Brussels, Belgium, (2002).

[ref58] Reis J. A. Paula A. T. Casarotti S. N. Penna A. L. B. (2012). Lactic acid bacteria antimicrobial compounds: characteristics and applications. Food Eng. Rev. 4, 124–140. doi: 10.1007/s12393-012-9051-2

[ref59] Sabbagh N. Gheisari H. R. Aminlari M. (2010). Monitoring the chemical and microbiological changes during ripening of Iranian probiotic low-fat white cheese. Am. J. Anim. Vet. Sci. 5, 249–257. doi: 10.3844/ajavsp.2010.249.257

[ref60] Sarantinopoulos P. Kalantzopoulos G. Tsakalidou E. (2002). Effect of *Enterococcus faecium* on microbiological, physicochemical and sensory characteristics of Greek feta cheese. Int. J. Food Microbiol. 76, 93–105. doi: 10.1016/S0168-1605(02)00021-1, PMID: 12038582

[ref61] Seixas F. N. Rios E. A. Martinez de Oliveira A. L. Beloti V. Poveda J. M. (2018). Selection of Leuconostoc strains isolated from artisanal serrano Catarinense cheese for use as adjuncts in cheese manufacture. J. Sci. Food Agric. 98, 3899–3906. doi: 10.1002/jsfa.8907, PMID: 29364508

[ref62] Server-Busson C. L. A. I. R. E. Foucaud C. LEVEAU J. Y. (1999). Selection of dairy Leuconostoc isolates for important technological properties. J. Dairy Res. 66, 245–256. doi: 10.1017/S0022029999003374

[ref63] Silva H. L. A. Balthazar C. F. Silva R. Vieira A. H. Costa R. G. B. Esmerino E. A.. (2018). Sodium reduction and flavor enhancer addition in probiotic Prato cheese: contributions of quantitative descriptive analysis and temporal dominance of sensations for sensory profiling. J. Dairy Sci. 101, 8837–8846. doi: 10.3168/jds.2018-14819, PMID: 30077456

[ref64] Skandamis P. N. Nychas G. J. (2001). Effect of oregano essential oil on microbiological and physico-chemical attributes of minced meat stored in air and modified atmospheres. J. Appl. Microbiol. 91, 1011–1022. doi: 10.1046/j.1365-2672.2001.01467.x, PMID: 11851808

[ref65] Skeie S. Lindberg C. Narvhus J. (2001). Development of amino acids and organic acids in Norvegia, influence of milk treatment and adjunct Lactobacillus. Int. Dairy J. 11, 399–411. doi: 10.1016/S0958-6946(01)00075-9

[ref66] Terpou A. Bekatorou A. Bosnea L. Kanellaki M. Ganatsios V. Koutinas A. A. (2018a). Wheat bran as prebiotic cell immobilisation carrier for industrial functional feta-type cheese making: chemical, microbial and sensory evaluation. Biocatal. Agric. Biotechnol. 13, 75–83. doi: 10.1016/j.bcab.2017.11.010

[ref67] Terpou A. Mantzourani I. Galanis A. Kanellaki M. Bezirtzoglou E. Bekatorou A.. (2018b). Employment of *L. paracasei* K5 as a novel potentially probiotic freeze-dried starter for feta-type cheese production. Microorganisms 7:3. doi: 10.3390/microorganisms7010003, PMID: 30587786 PMC6352075

[ref68] Tripathi M. K. Giri S. K. (2014). Probiotic functional foods: survival of probiotics during processing and storage. J. Funct. Foods 9, 225–241. doi: 10.1016/j.jff.2014.04.030

[ref69] Yousefi L. Dovom M. R. E. Najafi M. B. H. Mortazavian A. M. (2021). Antioxidant activity of ultrafiltered-feta cheese made with adjunct culture during ripening. J Food Measurement and Characterization 15, 4336–4342. doi: 10.1007/s11694-021-01019-0

