# Supplementary material for: Application of multi-functional lactic acid bacteria strains in a pilot scale feta cheese production
Source: Front Microbiol. 2023 Oct 11;14:1254598. doi: 10.3389/fmicb.2023.1254598 (PMC10598639; doi:10.3389/fmicb.2023.1254598)
Supplement: Supplementary file 1 [file Data_Sheet_1.docx]

Supplementary Material

**Application of multi-functional lactic acid bacteria strains in a pilot scale Feta cheese production**

Christina S. Kamarinou^1,2^, Olga S. Papadopoulou^1^, Agapi I. Doulgeraki^1^, Chrysoula C. Tassou^1^, Alex Galanis^2^, Nikos G. Chorianopoulos^3^ and Anthoula A. Argyri^1^*

*Corresponding author: [anthi.argyri@elgo.gr](mailto:anthi.argyri@elgo.gr)

# Supplementary Tables and Figures

##
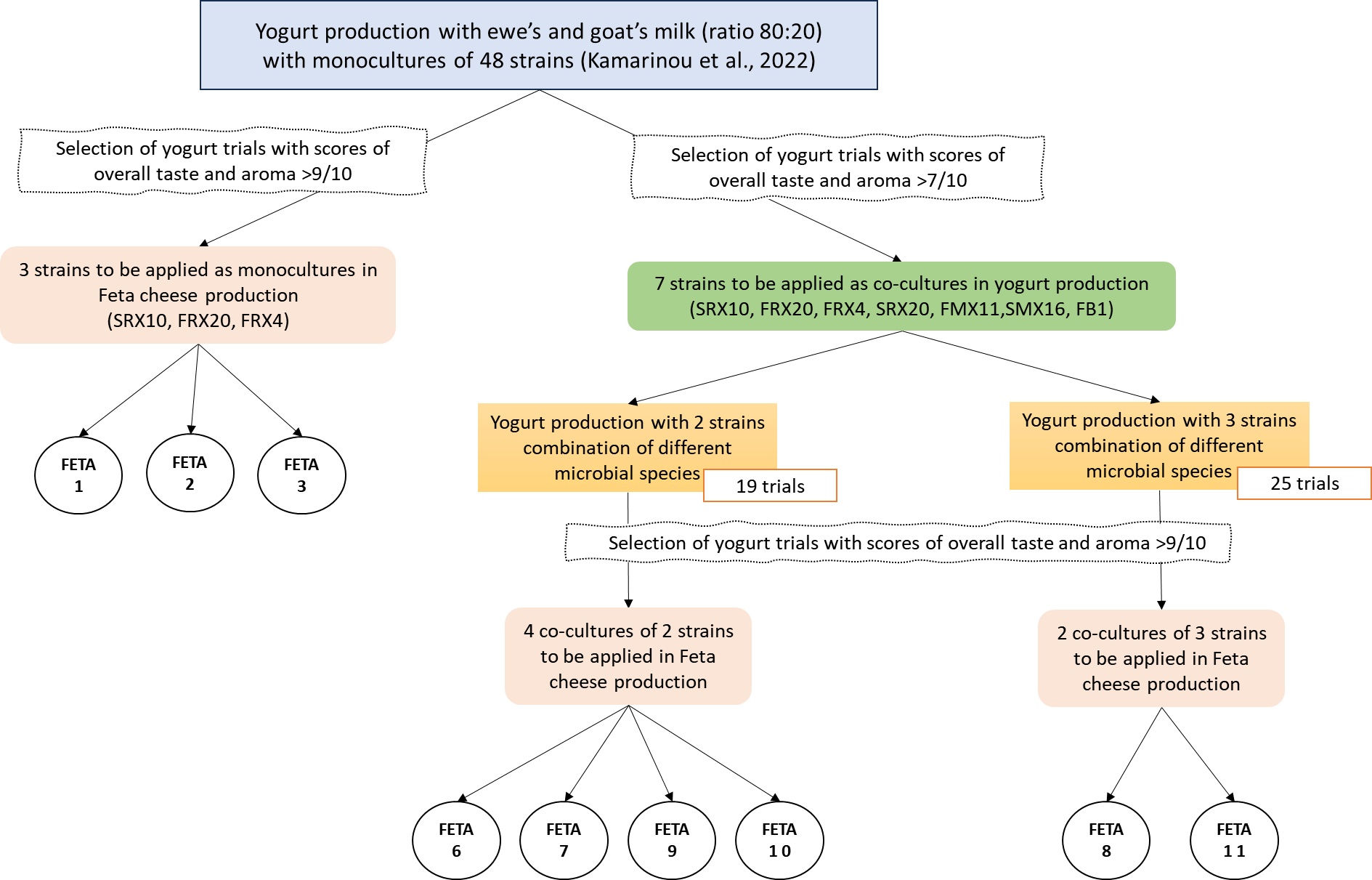


## Supplementary Figure 1: Preliminary experiments to select mono or co-cultures of LAB strains for Feta cheese production, according to their contribution to yogurt sensory profile. The Feta cheese trials FETA 4, 5 and 12 that are not included in the graph were produced with mono and co-cultures of the strains SMX2 and FMX3, due to their anti-listerial properties.


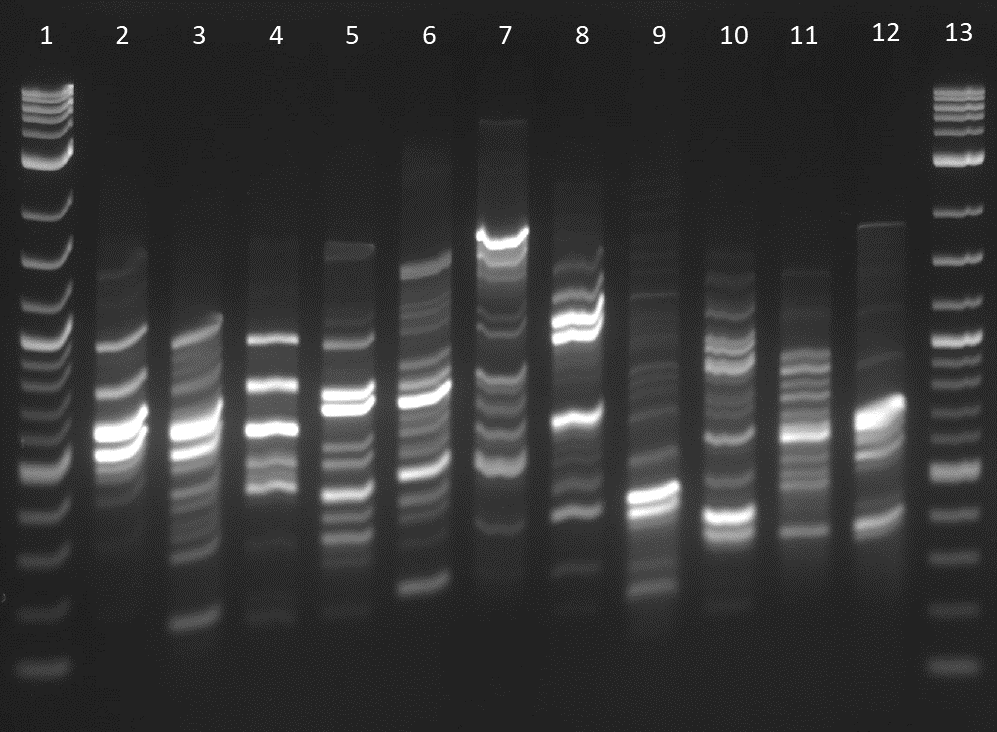


Supplementary Figure 2: RAPD-PCR fingerprinting profiles using the M13 primer. Lane 1 and 13: Ladder with molecular size marker (1 kbp ladder), Lane 2: FRX4 isolate, Lane 3: FMX3 isolate, Lane 4: FMX11 isolate, Lane 5: SRX10 isolate, Lane 6: SRX20 isolate, Lane 7: SMX2 isolate, Lane 8: SMX16 isolate, Lane 9: FRX20 isolate, Lane 10: FB1 isolate, Lane 11 and Lane 12: Isolations from the starter culture (1D and 2D).

Supplementary Table 1: Physicochemical characteristics (salt, protein, fat and moisture contents of cheese [g/100 g of cheese]) of the 13 Feta cheese trials at the beginning (day 60) and at the end of storage (day 180).

| Feta trial | Salt% | | Protein % | | Fat % | | Moisture % | |
| --- | --- | --- | --- | --- | --- | --- | --- | --- |
|  | **60d** | **180d** | **60d** | **180d** | **60d** | **180d** | **60d** | **180d** |
| Feta 1 | 2.73 | 2.55 | 13.82 | 14.84 | 20.89 | 23.36 | 59.24 | 56.08 |
| Feta 2 | 2.76 | 2.39 | 15.33 | 15.44 | 24.14 | 23.99 | 54.98 | 55.24 |
| Feta 3 | 2.57 | 2.49 | 14.73 | 16.01 | 22.75 | 25.36 | 56.85 | 53.77 |
| Feta 4 | 2.88 | 2.45 | 14.81 | 15.42 | 24.17 | 24.26 | 55.38 | 54.89 |
| Feta 5 | 2.95 | 2.56 | 14.35 | 15.45 | 23.85 | 24.16 | 55.88 | 54.81 |
| Feta 6 | 2.74 | 2.56 | 15.92 | 15.32 | 25.50 | 24.21 | 53.35 | 55.28 |
| Feta 7 | 2.83 | 2.60 | 14.81 | 14.86 | 24.11 | 24.29 | 55.27 | 55.42 |
| Feta 8 | 2.73 | 2.95 | 15.75 | 14.91 | 24.88 | 24.84 | 54.18 | 54.62 |
| Feta 9 | 2.82 | 2.41 | 14.39 | 15.71 | 23.24 | 24.36 | 56.83 | 54.74 |
| Feta 10 | 2.55 | 2.57 | 16.01 | 15.21 | 24.96 | 24.62 | 53.92 | 55.10 |
| Feta 11 | 2.57 | 2.47 | 15.89 | 16.29 | 24.68 | 25.27 | 54.07 | 53.18 |
| Feta 12 | 2.87 | 2.55 | 14.67 | 16.79 | 23.93 | 26.62 | 55.86 | 51.80 |
| Feta 13 | 2.65 | 2.67 | 15.72 | 14.88 | 24.66 | 23.87 | 54.20 | 55.72 |


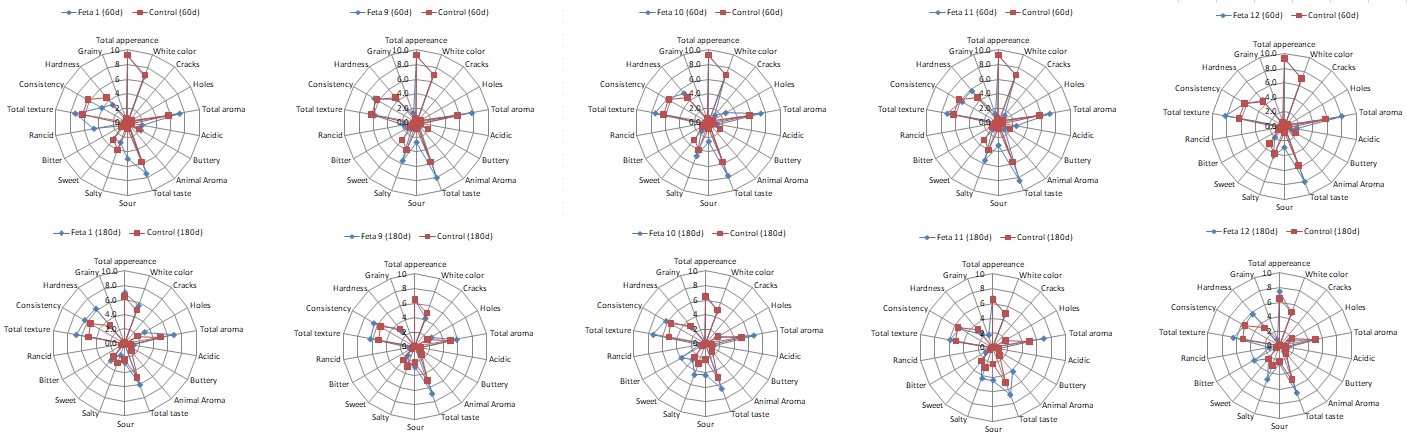


Supplementary Figure 3: Sensory evaluation of selected Feta trials without (♦) (control) and with (■) adjunct cultures (Feta 1, 9, 10, 11 and 12), at day 60 and day 180 after cheese production stored at 4℃


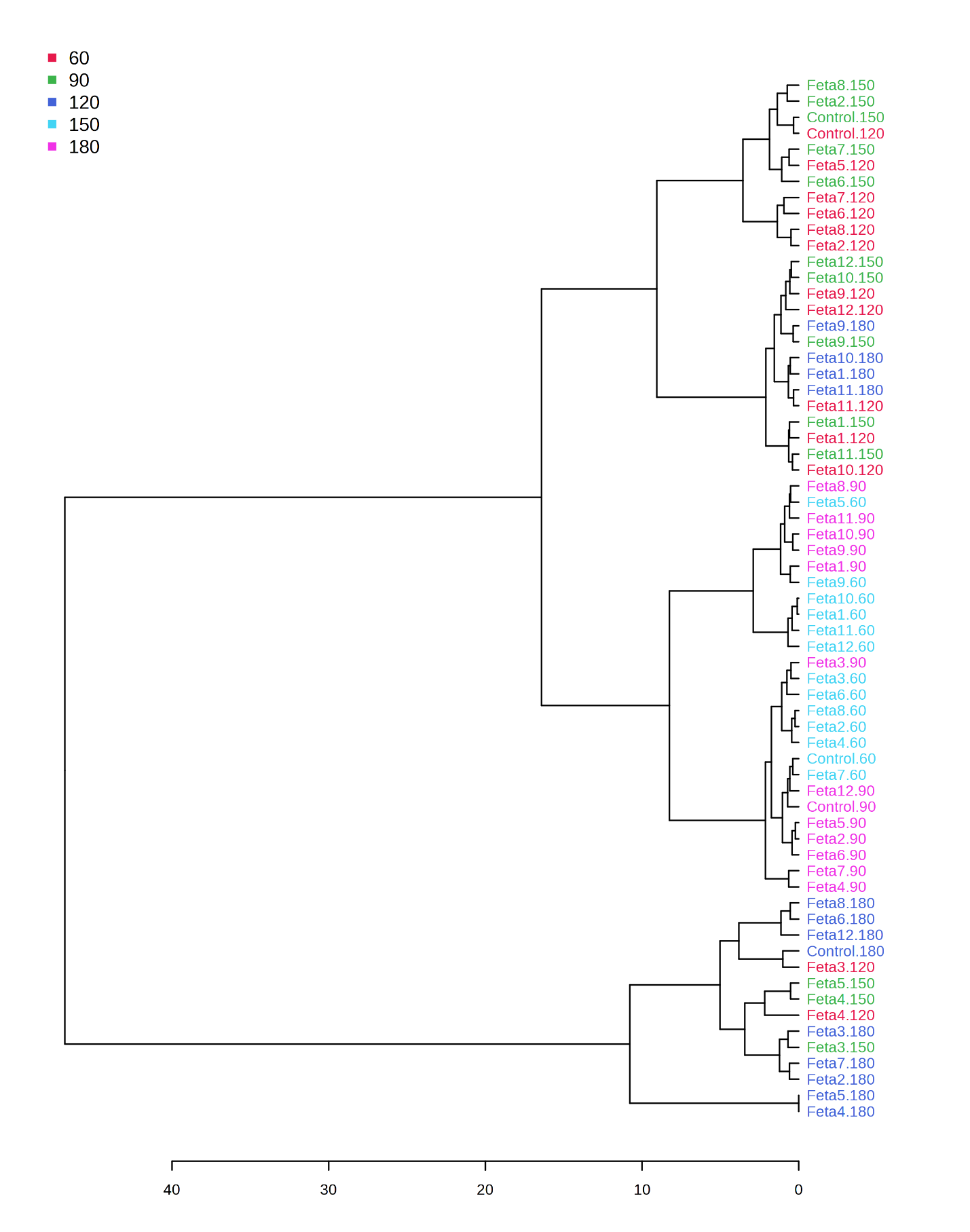


Supplementary Figure 4: Hierarchical clustering result shown as a dendrogram of sensory scores associated with the different storage days of the 12 Feta cheese trials. Ward-linkage clustering was based on the Euclidean correlation coefficients.


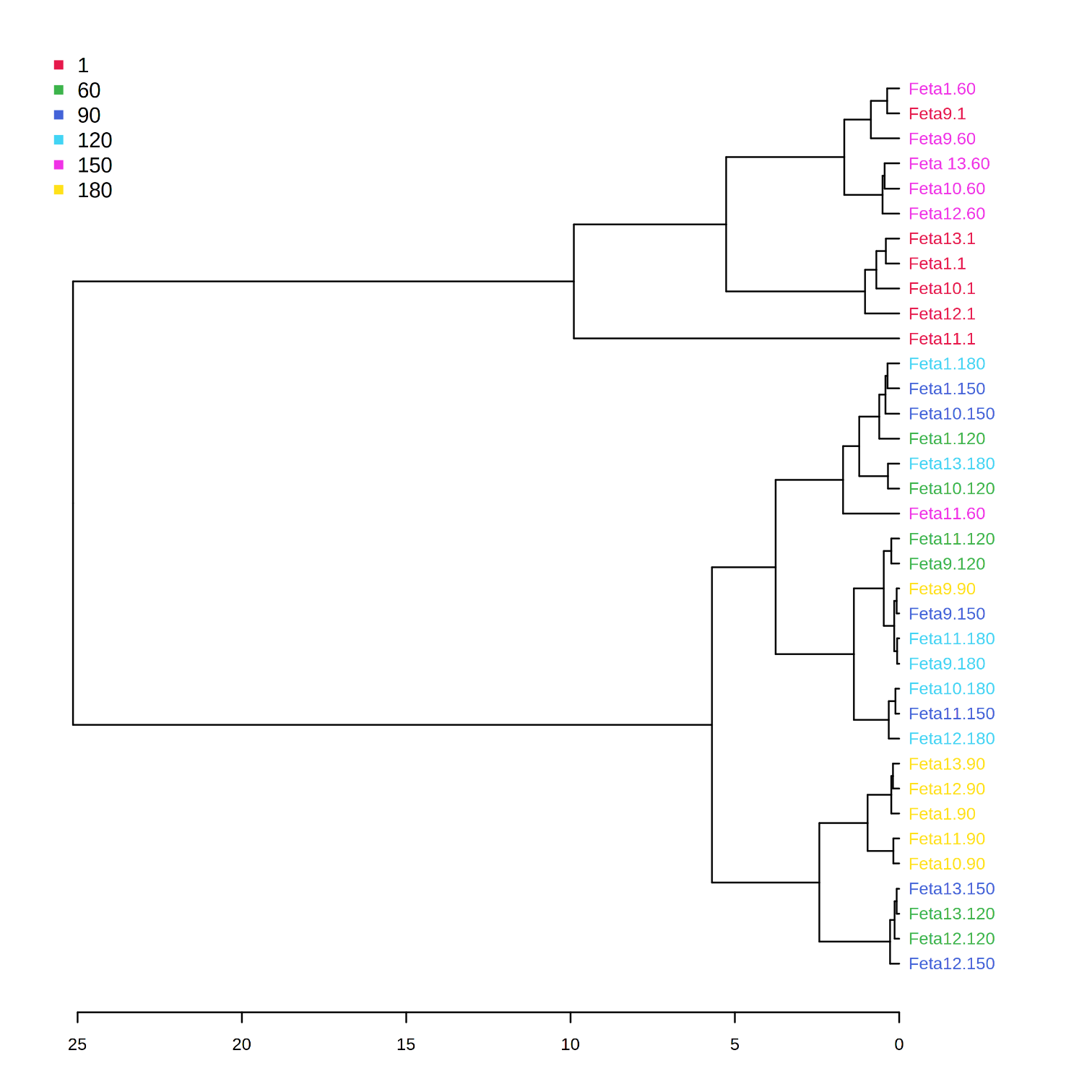


Supplementary Figure 5: Hierarchical clustering result shown as a dendrogram of organic acids associated with the different days (ripening and storage) of the 6 Feta cheese trials. Ward-linkage clustering was based on the Euclidean correlation coefficients.
